# Blood-based Vienna 3P/5P risk models accurately predict first hepatic decompensation in compensated advanced chronic liver disease^☆^

**DOI:** 10.1016/j.jhepr.2025.101642

**Published:** 2025-10-17

**Authors:** Georg Kramer, Benedikt Simbrunner, Mathias Jachs, Lorenz Balcar, Benedikt Silvester Hofer, Nina Dominik, Lukas Hartl, Michael Schwarz, Georg Semmler, Christian Sebesta, Paul Thöne, Sophia Geisselbrecht, Benjamin Maasoumy, Eduardo Alvarez, Martin Sebastian McCoy, Oleksandr Petrenko, Jiří Reiniš, Philipp Schwabl, Albert F. Stättermayer, Michael Trauner, Mattias Mandorfer, Thomas Reiberger

**Affiliations:** 1 Division of Gastroenterology and Hepatology, Department of Medicine III, Medical University of Vienna, Vienna, Austria; 2 Vienna Hepatic Hemodynamic Laboratory, Division of Gastroenterology and Hepatology, Department of Medicine III, Medical University of Vienna, Vienna, Austria; 3 Clinical Research Group MOTION, Medical University of Vienna, Vienna, Austria; 4 Christian-Doppler Laboratory for Portal Hypertension and Liver Fibrosis, Medical University of Vienna, Vienna, Austria; 5 Department of Gastroenterology, Hepatology, Infectious Diseases and Endocrinology, Hannover Medical School Hannover, Germany; 6 Department of Internal Medicine B, University Hospital Muenster (UKM), Muenster, Germany; 7 CeMM Research Center for Molecular Medicine of the Austrian Academy of Sciences, Vienna, Austria; 8 Department of Laboratory Medicine, Medical University Vienna, Vienna, Austria; 9 Ukrainian Institute for Systems Biology and Medicine, 04119 Kyiv, Ukraine

**Keywords:** Cirrhosis, Baveno, clinically significant portal hypertension, non-invasive tests, machine learning, betablockers, liver stiffness

## Abstract

**Background & Aims:**

Invasive measurement of hepatic venous pressure gradient (HVPG) is the gold standard for diagnosing clinically significant portal hypertension (CSPH, *i.e.* HVPG ≥10 mmHg), which indicates an increased risk of decompensation. We evaluated the blood-based Vienna 3P/5P models for non-invasive assessment of portal hypertension (PH) severity and their prognostic value. Their performance was compared to HVPG, liver stiffness measurement (LSM) and the ANTICIPATE±NASH model.

**Methods:**

Patients with compensated advanced chronic liver disease (cACLD) who underwent HVPG measurement and LSM within the prospective VICIS (Vienna Cirrhosis Study) were included. We assessed the ability of each model to detect CSPH and severe PH (HVPG ≥16 mmHg), predict decompensation, and stratify risk. Outcome prediction was further validated in an external cohort.

**Results:**

Among 266 patients with diverse etiologies of cACLD, median HVPG was 11 (8-16) mmHg with a CSPH and severe PH prevalence of 62.8% and 25.6%, respectively. The 3P/5P models correlated with HVPG (both *p <*0.001), achieving AUROCs of 0.704 (5P) for CSPH and 0.800 (5P) for severe PH prediction. During a median follow-up of 23.9 (15.3-32.6) months, 48 (18%) patients decompensated. HVPG and the 5P model showed similar time-dependent AUROCs (at 0.5 and 1 year: 0.753-0.822), superior to ANTICIPATE±NASH (AUROCs: 0.689-0.691) and LSM (AUROCs: 0.621-0.636). The 5P (adjusted subdistribution hazard ratio [aSHR]: 1.32, *p<*0.001) and 3P (aSHR: 1.15, *p =* 0.010) models predicted decompensation independent from age, sex, LSM, etiological cure and non-selective beta blocker use. Proposed cut-offs for the 3P/5P models distinguished between patients at low and high risk of decompensation (Grays test *p <*0.001).

**Conclusion:**

The blood-based 3P/5P models demonstrated significant prognostic value for predicting hepatic decompensation and identifying patients with cACLD at high risk. Importantly, the 5P model performed comparably to HVPG.

**Impact and implications:**

This study addresses the clinical need for accessible, reliable, and cost-effective non-invasive tools to predict hepatic decompensation in patients with compensated advanced chronic liver disease, given the limited availability of hepatic venous pressure gradient and liver stiffness measurement. By demonstrating that the Vienna 3P/5P models – machine learning tools based solely on routine laboratory parameters – achieve comparable prognostic accuracy to hepatic venous pressure gradient and outperform other non-invasive tools, such as liver stiffness measurement or the ANTICIPATE±NASH model, these findings have significant implications for clinicians providing care for patients with compensated advanced chronic liver disease. The models' simplicity, repeatability and wide availability could facilitate timely risk stratification and improved clinical management across diverse healthcare settings.

**Clinical trial number:**

NCT03267615.

## Highlights

• Blood-based 3P/5P machine learning models predict decompensation in cACLD.
• The 5P model has similar prognostic value as invasive HVPG measurement.
• Both models perform favourably against LSM and ANTICIPATE±NASH.
• 3P/5P models might support individualized prognostication and treatment in cACLD.

## Graphical abstract

**Figure:**
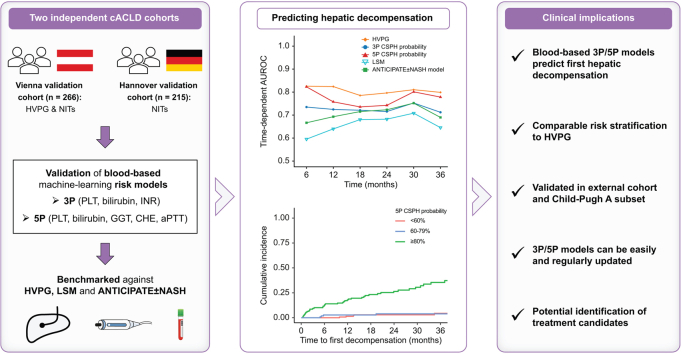

## Introduction

Portal hypertension (PH) is the main driver of complications in compensated advanced chronic liver disease (cACLD).^1^ While measurement of the hepatic venous pressure gradient (HVPG) is considered the gold standard for assessing PH, it is an invasive procedure requiring specialized equipment and profound expertise, limiting its availability in clinical routine.^2^ Clinically significant portal hypertension (CSPH) is defined by an HVPG ≥10 mmHg and identifies patients at risk of hepatic decompensation,^3^^,^^4^ while an HVPG of ≥16 mmHg, indicating severe PH, is associated with an imminent risk of disease progression and complications.[5], [6], [7]

Early diagnosis of CSPH, combined with timely treatment using non-selective beta blockers (NSBBs), can significantly reduce the risk of variceal bleeding, hepatic decompensation and liver-related mortality.[8], [9], [10] As a result, significant efforts have been directed toward developing and validating non-invasive tests (NITs) for identifying CSPH in clinical practice, aiming to provide accurate, accessible and cost-effective alternatives to HVPG measurement.^2^^,^^11^

The recent Baveno VII consensus^2^ recommends a NIT algorithm combining liver stiffness measurement (LSM) via vibration-controlled transient elastography (VCTE) and platelet count (PLT) based on the findings of the ANTICIPATE study^12^. Moreover, the ANTICIPATE- or ANTICIPATE±NASH^13^ (the latter considering BMI) models, as applicable, are suggested for predicting CSPH risk. However, applying these criteria leaves approximately 50% of patients with cACLD in a diagnostic ‘grey zone’.[14], [15], [16]

To reduce the proportion of unclassified patients, alternative/sequential algorithms have been proposed, including utilizing spleen stiffness measurement (SSM)^15^ or the von Willebrand factor (vWF) to platelet ratio (VITRO).^16^^,^^17^ However, vWF is not routinely measured and can vary widely across different assay platforms. As a result, vWF-based parameters remain experimental, necessitating further prognostic validation and standardized conversion factors. Meanwhile, other more established blood-based tests such as the fibrosis-4 (FIB-4) score^18^ have been designed for broad fibrosis screening in primary care rather than specifically for CSPH detection. Finally, machine learning models (MLMs) have been developed to predict HVPG using liver biopsy data and laboratory parameters, although these models still rely on invasive biopsies.^19^

To address these limitations, the Vienna MLMs that are solely based on either three (3P) or five (5P) routine laboratory values have recently been introduced to predict CSPH and severe PH.^20^ These models have shown promising results in initial studies, yielding good diagnostic accuracy for CSPH and severe PH in external validation cohorts of various etiologies of cACLD.^20^^,^^21^

Despite the promising discriminative capabilities of the Vienna 3P/5P models in patients with cACLD, its lack of validation in further cohorts and for predicting clinical outcomes, such as hepatic decompensation, development of hepatocellular carcinoma (HCC) or liver-related mortality leaves a significant gap in understanding their generalizability and applicability.

The primary aim of this study was to evaluate the prognostic performance of the Vienna 3P and 5P models for clinical outcomes in a heterogeneous cohort of patients with cACLD. As a secondary objective, we validated the models’ ability to predict the severity of PH. We further compared the performance of these models to invasive HVPG-based risk assessment as well as established NITs such as LSM and the Baveno VII-suggested ANTICIPATE±NASH model. Outcome prediction was moreover analyzed in an external validation cohort from Hannover.

## Patients and methods

### Patient selection and study design

Patients with cACLD, defined as HVPG ≥6 mmHg, LSM ≥10 kPa, or F3/F4 fibrosis on histology, without prior or current hepatic decompensation who underwent characterization by same-day HVPG and NIT assessment at the Vienna Hepatic Hemodynamic Lab and have been recruited in the prospective Vienna Cirrhosis Study (VICIS, NCT03267615) between 2017 and 2023 were considered for this study. Exclusion criteria included: (i) previous or current hepatic decompensation; (ii) unreliable HVPG measurement (presence of severe intrahepatic veno-venous communications, technical complications), (iii) measurement during non-selective beta-blocker (NSBB) intake, (iv) presence of occlusive portal venous thrombosis, vascular or posthepatic/cardiac liver disease; (v) hepatic or active extrahepatic malignancies; (vi) previous liver transplantation (LT); and (vii) acute liver failure at the time of HVPG measurement.

Out of the overall Vienna cohort, the HVPG prediction cohort and the outcome prediction cohort were formed. Specifically, the HVPG prediction cohort was established by excluding patients from the overall cohort who had been part of the training set used in the initial development and publication of the Vienna 3P/5P models.^20^ The outcome prediction cohort was created by removing patients without sufficient follow-up data/duration from the overall cohort. This process yielded two partially overlapping cohorts: out of the overall 266 included patients, 6 patients were exclusively assigned to the HVPG prediction cohort, 125 patients were shared between both cohorts, and 135 patients were only included in the outcome prediction cohort. A flowchart illustrating the cohort formation and patient exclusions is provided in Fig. S1.

Furthermore, sub-cohorts consisting exclusively of patients with Child-Turcotte-Pugh Stage A (CTP A) from both the HVPG prediction cohort and the outcome prediction cohort were created for sensitivity analysis. The respective sub-cohorts are hereafter referred to as HVPG prediction CTP A sub-cohort (n = 101) and outcome prediction CTP A sub-cohort (n = 219), respectively.

The primary endpoint was the onset of first hepatic decompensation (ascites, overt hepatic encephalopathy, or variceal bleeding). Performance of LT, development of HCC, liver-related and non-liver-related death were recorded and considered for competing risk analyses. Patients who did not experience these events were censored at the date of their last recorded clinical contact. Follow-up data were collected through a thorough manual review of individual medical records, supplemented by queries into national electronic health databases.

### Achievement of etiological cure

In accordance with the Baveno VII criteria,^2^ achievement of etiological cure was defined as removal of the primary etiological factor. Specifically, this included: (i) documented sustained alcohol abstinence in alcohol-related liver disease, (ii) sustained virological response in HCV-associated cirrhosis, and (iii) suppression of viral replication in HBV-associated cirrhosis under treatment with nucleos(t)ide analogs. Alcohol intake was assessed at each outpatient visit and inpatient stay using a combination of patient and/or caregiver reports given within a structured interview and the treating hepatologist’s clinical judgment. In patients with more than one etiology, etiological cure was assumed only when all causative factors had been removed or suppressed.

### HVPG measurement and LSM

HVPG measurements were performed under fasting conditions, adhering to established guidelines.^2^^,^^22^ Following local anesthesia and central venous access via an internal jugular vein, a suitable hepatic vein was cannulated, and free and wedged hepatic venous pressures were measured at least in triplicate. HVPG was calculated as the difference between wedged hepatic venous pressure and free hepatic venous pressure, with the final value being the mean of the three measurements. LSM was measured using VCTE with the FibroScan® device (Echosense, Paris, France), following established protocols as previously reported.^23^^,^^24^

### Assessment of routine laboratory parameters

Routine laboratory tests and biomarker analyses were performed by the ISO-certified Department of Laboratory Medicine at the Medical University of Vienna, using commercially approved methods for clinical use and blood sample analysis.

### Non-invasive HVPG prediction and CSPH risk assessment

HVPG prediction and CSPH probability estimations were calculated using the Vienna 3P/5P models, based on their respective published formulas.^20^ To ensure comparability with LSM and the ANTICIPATE model, we did not evaluate the probability output for severe PH from the 3P/5P models in this study. The 3P model incorporates platelets (PLT), bilirubin, and international normalized ratio, while the 5P model includes PLT, bilirubin, activated partial thromboplastin time, cholinesterase and gamma-glutamyltransferase. The ANTICIPATE model’s formula^12^ utilizes LSM and PLT to estimate the probability of CSPH, while the ANTICIPATE-NASH model, applied in patients with obese metabolic dysfunction-associated steatotic liver disease (MASLD)/metabolic- and alcohol-related liver disease (MetALD) additionally incorporates BMI.^13^ This combined approach is hereafter referred to as the ANTICIPATE±NASH model. We deliberately excluded SSM and SSM-derived NITs, such as the recently proposed NICER model,^25^ from our comparative analyses. Although these methods demonstrate considerable promise with SSM being increasingly established in clinical practice, current guidelines^2^ do not yet recommend its use for non-viral etiologies. Moreover, SSM was not routinely performed at our center during the study period, with data available for only 18% of patients, precluding robust and unbiased comparisons.

### Algorithms for ruling in or ruling out CSPH and according to risk stratification for first hepatic decompensation

Per the Baveno VII consensus,^2^ CSPH was ruled out if PLT ≥150 G/L and LSM ≤15 kPa, while CSPH was ruled in if LSM ≥25 kPa. The cohort was further stratified into risk groups by HVPG (<10 mmHg, 10-15 mmHg, ≥16 mmHg) and by specified NITs. As reliable thresholds for ruling in CSPH using the Vienna 3P and 5P models have not yet been established,^26^^,^^27^ and were previously only proposed for the 3P model,^20^ optimal thresholds based on Youden’s index were calculated using the ‘pROC’-package (v.11.18.5).^28^ Additionally, cut-offs achieving 90% specificity for ruling in or 90% sensitivity for ruling out CSPH were calculated for the Vienna 3P/5P models. Based on these results, cut-offs of <60% or <10 mmHg to rule out and of ≥80% or ≥15 mmHg to rule in CSPH were selected for outcome prediction analysis. For the ANTICIPATE±NASH model, the Baveno VII implied 60% cut-off, which had previously also been proposed for linear risk stratification models, was used.

### External validation cohort

An external validation cohort of patients from the Hannover Medical School was included, applying the same inclusion and exclusion criteria as in the Vienna cohorts. This external cohort was used to validate the prognostic performance of the Vienna 3P/5P models.

### Statistical analysis

Statistical analyses were conducted using R 4.4.0 (The R Foundation, Vienna, Austria). Continuous variables are reported as mean ± SD or median (IQR), depending on data normality, based on normality assessed via normality plots, Q-Q plots and the Shapiro-Wilk test. Comparisons of normally distributed variables were performed using Student’s *t* test with Mann-Whitney *U* test being used for non-normally distributed variables. The correlation between two continuous variables was assessed using Spearman’s rank correlation coefficient. Group comparisons of categorical variables were performed using either Pearson’s chi-squared or Fisher’s exact test, as applicable.

The AUROC and corresponding 95% CIs were calculated and compared by DeLong tests using the ‘pROC’-package. Calibration plots with bootstrapping (1,000 repetitions) were generated using the ‘rms’-package (v.6.8.1).^29^

Cumulative incidences were calculated and compared using Gray’s test with the ‘cmprsk’-package (v.2.2.12),^30^ which was also used to compute univariable and multivariable Fine-Gray competing risk regression models. Separate multivariable models were fitted for each individual predictor of hepatic decompensation and adjusted for relevant covariates: age, sex, LSM, achievement of etiological cure and NSBB intake. Importantly, the models incorporating the ANTICIPATE±NASH model were not adjusted for LSM to avoid overfitting, as LSM is a core component of this model. In contrast to the 3P/5P models, which incorporate liver function markers, the ANTICIPATE±NASH model required adjustment for baseline liver function, for which albumin was used instead of LSM. In each Fine-Gray model, we confirmed the proportional-subdistribution hazards assumption for each predictor by computing modified weighted Schoenfeld residuals via the ‘crrSC’-package (v1.1.2)^31^ and evaluated multicollinearity between model parameters via variance inflation factor analysis using the ‘car’-package (v 3.1-3).^32^

For analysis of first hepatic decompensation, competing events were HCC development and non-liver-related death. For liver-related mortality, competing events included non-liver-related death and LT, while for HCC occurrence, all-cause death and LT were treated as competing risks.

Calculation and multiple-testing-adjusted comparison of time-dependent AUROCs was conducted via the ‘timeROC’-package (v.0.4). Detailed methodology is provided in the original work by Blanche *et al.*,^33^ on which the package is based.

A two-sided *p* value <0.05 was considered statistically significant across all analyses.

### Ethical aspects

This study was conducted in accordance with the principles of the 1964 Helsinki Declaration and its later amendments and received approval from the local ethics committee of the Medical University of Vienna (EK 1262/2017).

## Results

### Patient characteristics of the Vienna cohort

A total of 266 patients with cACLD were included in the study. The predominant etiology was viral liver disease, affecting 27.8% of patients, followed by alcohol-related liver disease in 23.7% and MASLD in 17.7%. Median HVPG was 11 mmHg (IQR: 8-16 mmHg), with CSPH present in 62.8% and severe PH in 25.6% of cases. Median VCTE-LSM was 22.3 kPa (IQR: 15.5-37.0 kPa). Esophageal varices were detected in 38.8% of patients. Further details on patient characteristics are provided in Table 1 and Table S1.

The HVPG prediction cohort and the outcome prediction cohort differed only in the higher prevalence of severe PH in the latter (25.8% *vs.* 16%) (Table 1). However, the number of patients diagnosed with CSPH, as well as median NIT and laboratory values, did not differ significantly between the cohorts.

**Table 1:** Patient characteristics.

	Vienna overall cohort	Hannover cohort	*p* value
Participants, n	266	215	
Age, years, mean (SD)	55.40 (12.22)	57.34 (11.01)	0.072
Sex, n (%)			0.127
Male	175 (65.8)	126 (58.6)	
Female	91 (34.2)	89 (41.4)	
BMI, kg/m^2^ (IQR)	26.9 (23.5, 30.6)	26.4 (23.7, 29.8)	0.615
Etiology, n (%)			**<0.001**
ALD	64 (24.1)	0 (0.0)	
Viral hepatitis	74 (27.8)	210 (97.7)	
ALD + viral hepatitis	15 (5.6)	0 (0.0)	
MASLD	47 (17.7)	3 (1.4)	
Cholestatic	23 (8.6)	1 (0.5)	
Other	43 (16.2)	1 (0.5)	
Achievement of etiological cure, n (%)	108 (41.7)	210 (97.7)	**<0.001**
HVPG, mmHg, median (IQR)	10 (7-13.5)	-	
CSPH, n (%)	167 (62.8%)	-	
Severe PH, n (%)	68 (25%)	-	
Varices			0.416
None, n (%)	153 (61.2)	57 (65.5)	
Small, n (%)	69 (27.6)	18 (20.7)	
Large, n (%)	28 (11.2)	12 (13.8)	
NSBB intake, n (%)	143 (57.4%)	NA	NA
Child-Turcotte-Pugh score	5.00 (5.00, 6.00)	5.00 (5.00, 6.00)	0.547
VCTE-LSM, kPa median (IQR)	22.3 (15.5, 37.0)	19 (14.1, 28.0)	**0.007**
ANTICIPATE±NASH CSPH probability (%)	69.83 (38.25, 92.55)	55.00 (28.03, 88.42)	**0.025**
Vienna 3P model			
CSPH probability (%)	76.18 (62.93, 87.02)	62.89 (48.24, 75.51)	**<0.001**
Predicted HVPG, mmHg (IQR)	13.77 (12.30, 15.09)	12.51 (10.95, 13.78)	**<0.001**
Vienna 5P model			
CSPH probability (%)	74.8 (57.9, 87.3)	57.16 (31.66, 81.27)	**<0.001**
Predicted HVPG, mmHg (IQR)	13.4 (11.6, 15.6)	10.87 (8.69, 13.65)	**<0.001**
MELD, median (IQR)	9 (8, 12)	8.00 (7, 9)	**<0.001**
Platelet count, G/L, median (IQR)	110 (83, 160)	130 (96, 180)	**0.002**
Bilirubin, mg/dl, median (IQR)	0.92 (0.63, 1.53)	0.70 (0.47, 0.94)	**<0.001**
INR, median (IQR)	1.2 (1.1, 1.4)	1.10 (1.1, 1.2)	**<0.001**
aPTT, s, median (IQR)	38.20 (35.20, 41.65)	32.00 (30.00, 37.00)	**<0.001**
Albumin, g/L, median (IQR)	39.05 (36.02, 41.68)	38.00 (35.00, 41.00)	0.147
CHE, kU/L, median (IQR)	5.39 (4.07, 6.93)	6.40 (4.58, 7.91)	**<0.001**
GGT, U/L, median (IQR)	96 (47, 187)	93 (59, 159)	0.942

Data expressed as n (%), mean (SD) or median (IQR). Continuous variables were analyzed using either an independent samples *t* test or Mann-Whitney *U* test, depending on distribution. Categorical variables were compared using Pearson’s Chi-squared test or Fisher’s exact test. In the Vienna overall cohort LSM and ANTICIPATE±NASH CSPH probability data were unavailable for 9 (3.4%) and 11 (4.2%) patients, respectively, with information on etiological cure missing in 7 (2.6%) patients. In the Hannover cohort, aPTT was missing in 65 (30.2%) patients and cholinesterase in 1 (0.05%) patient; thus, data for the 5P model was missing in 66 (37.5%) patients. *P* values in bold indicate statistical significance (*p* <0.05). ALD, alcohol-related liver disease; aPTT, activated partial thromboplastin time; CHE, cholinesterase; CSPH, clinically significant portal hypertension; GGT, gamma-glutamyltransferase; HVPG, hepatic venous pressure gradient; INR, international normalized ratio; MASLD, metabolic dysfunction-associated steatotic liver disease; MELD, model for end-stage liver disease; NSBB, non-selective beta-blocker; PH, portal hypertension; VCTE-LSM, vibration-controlled transient elastography liver stiffness measurement.

### Patient characteristics of the Hannover cohort

Using identical in- and exclusion criteria, we included an external validation cohort of 215 patients from the Hannover Medical School. Unlike the Vienna cohorts, the underlying liver disease in this external cohort was almost exclusively viral (97.7%); with every viremic patient receiving antiviral therapy or achieving a sustained virologic response. Overall, the Hannover cohort had less advanced liver disease, reflected by lower MELD (model for end-stage liver disease) scores (median 8 [IQR 7-9] *vs.* 9 [8-12]; *p <*0.001) and NITs such as LSM (19.0 [IQR 14.1-28.0] *vs.* 22.3 kPa [15.5-37.0]; *p =* 0.007).

### Vienna outcome prediction cohort

#### Cumulative incidence of liver-related events

During a median follow-up period of 23.9 months (IQR: 15.3-32.6 months), first hepatic decompensation occurred in 48 patients. LT was performed in 11 patients, 13 developed HCC, whereas 15 patients died from liver-related and 14 from non-liver-related causes. Moreover, 108 patients (41.7%) achieved etiological cure during the study period.

#### Prognostic capabilities of Vienna 3P/5P models and other NITs compared to HVPG

**Fig. 1:**
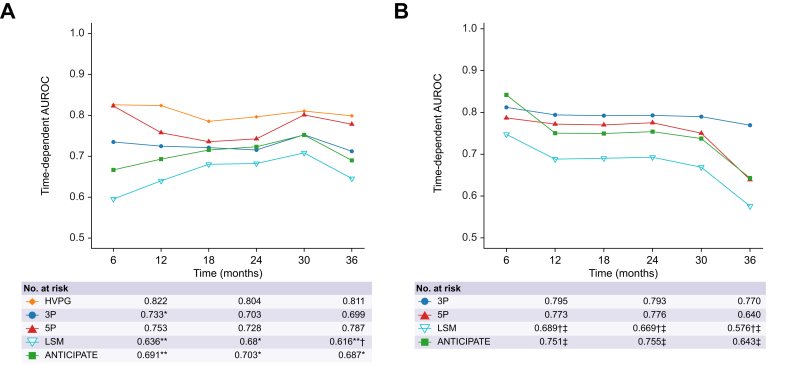
Time-dependent AUROCs for prediction of first hepatic decompensation in the Vienna outcome prediction and Hannover cohorts. Time-dependent AUROCs for prediction of first hepatic decompensation by the Vienna 3P/5P CSPH probability in the Vienna outcome prediction cohort (A) and the Hannover cohort (B) compared to HVPG, LSM and ANTICIPATE±NASH CSPH probability. Adjusted pairwise comparisons were performed via the ‘compare’-function of the ‘timeROC’-package. Levels of significance: ∗*p <*0.05 and ∗∗*p <*0.005 as compared to HVPG’s AUROC at the specified timepoint; ^†^*p <*0.05 as compared to one or both 5P Model’s AUROCs, ^‡^*p <*0.05 as compared to one or both of the 3P model’s AUROCs at the specified timepoint. CSPH, clinically significant portal hypertension; HVPG, hepatic venous pressure gradient; LSM, liver stiffness measurement; ROC, receiver operating characteristic.

HVPG demonstrated the highest time-depending AUROC for predicting short-term first hepatic decompensation at both 6 months and 1 year (AUROCs: 0.822). The Vienna 5P model showed comparable predictive performance for hepatic decompensation throughout a 3-year period, with exemplary AUROCs of 0.815 at 6 months and 0.753 at 1 year (Fig. 1).

For predicting hepatic decompensation at 6 months, the 5P CSPH probability yielded significantly higher AUROCs compared to the ANTICIPATE±NASH model (*p =* 0.034) and LSM (*p =* 0.021). Detailed results are shown in Fig. 1 and Table S6, with similar results observed in the outcome prediction CTP A sub-cohort, as illustrated in Fig. S6.

#### Risk factors for hepatic decompensation and liver-related mortality

When analyzing risk factors for first hepatic decompensation, HVPG (per mmHg, adjusted subdistribution hazard ratio [aSHR]: 1.21, *p <*0.001), the Vienna 5P model (predicted HVPG, per mmHg: aSHR: 1.32, *p <*0.001; CSPH probability, per 10%: aSHR: 1.42, *p =* 0.046), 3P model (predicted HVPG: aSHR: 1.15, *p =* 0.009; CSPH probability: aSHR: 1.15, *p =* 0.010) and the ANTICIPATE±NASH CSPH probability (per 10%; aSHR: 1.17, *p =* 0.038) were all independent predictors (Table 2).

**Table 2:** Risk factors for first hepatic decompensation.

	Univariable competing risk regression model			Multivariable competing risk regression models (adjusted for age, sex, LSM, achievement of etiological cure and NSBB intake)∗		
	SHR	95% CI	*p* value	aSHR	95% CI	*p* value
**VIENNA**						
Age, per year	0.99	0.96-1.01	0.310	-	-	-
Sex, male	1.01	0.55-1.84	0.980	-	-	-
LSM, per kPa	1.02	1.01-1.04	**0.002**	-	-	-
Albumin, per g/dl	0.92	0.87-0.96	**0.001**	-	-	-
Achievement of etiological cure, binary	0.49	0.26- 0.91	**0.024**	-	-	-
NSBB intake, binary	2.45	1.28-4.69	**0.007**	-	-	-
HVPG, per mmHg	1.16	1.11-1.22	**<0.001**	1.21	1.14-1.29	**<0.001**
3P CSPH probability, per 10%	1.48	1.10- 2.01	**0.011**	1.49	1.07-2.09	**0.019**
5P CSPH probability, per 10%	1.48	1.08-2.02	**0.014**	1.42	1.01-1.99	**0.046**
3P predicted HVPG, per mmHg	1.11	1.03-1.20	**0.008**	1.15	1.03-1.27	**0.010**
5P predicted HVPG, per mmHg	1.27	1.13-1.44	**<0.001**	1.32	1.13-1.55	**<0.001**
ANTICIPATE±NASH CSPH probability, per 10%∗	1.24	1.09-1.40	**<0.001**	1.17	1.01-1.37	**0.038**

**HANNOVER**						
Age, per year	1.03	0.97-1.08	0.370	-	-	-
Sex, male	1.60	0.50-5.14	0.430	-	-	-
LSM, per kPa	1.02	0.99-1.05	0.087	-	-	-
Albumin, per g/dl	0.94	0.88-0.99	**0.038**	-	-	-
Achievement of etiological cure, binary	0.13	0.02-0.93	**0.042**	-	-	-
NSBB intake, binary	8.40	1.18-59.90	**0.034**	-	-	-
3P CSPH probability, per 10%	1.65	1.07-2.57	**0.025**	1.65	0.89-3.07	0.120
5P CSPH probability, per 10%	1.80	1.36-2.39	**<0.001**	2.20	1.44-3.38	**<0.001**
3P predicted HVPG, per mmHg	1.48	1.10-2.00	**0.010**	1.54	0.92-2.59	0.100
5P predicted HVPG, per mmHg	1.25	1.13-1.38	**<0.001**	1.30	1.14-1.47	**<0.001**
ANTICIPATE±NASH CSPH probability, per 10%∗	1.55	1.25-1.92	**<0.001**	1.62	1.26-2.10	**<0.001**

Uni- and multivariable competing risk regression models assessing predictors for first hepatic decompensation. The multivariable models incorporate either HVPG, the Vienna 3P or 5P model or the ANTICIPATE±NASH model. Hepatocellular carcinoma diagnosis and non-liver-related death were treated as competing events. The results are presented as SHRs or aSHRs, along with 95% CIs and *p* values. *p* values in bold indicate statistical significance (*p* <0.05). aSHR, adjusted SHR; CRP, C-reactive protein; CSPH, clinically significant portal hypertension; HVPG, hepatic venous pressure gradient; LSM, liver stiffness measurement; NSBB, non-selective beta-blocker; SHR, subdistribution hazard ratio.

∗ The multivariable model incorporating the ANTICIPATE±NASH CSPH probability was adjusted to age, sex, achievement of etiological cure and albumin to avoid overfitting.

In multivariable analysis for liver-related death, HVPG (per mmHg; aSHR: 1.15, 95% CI 1.03-1.28, *p =* 0.011) was the sole independent predictor in our cohort. In univariable analysis, both HVPG and the MELD score were significant risk factors for liver-related mortality (Table S4).

#### Risk factors for HCC

In univariable analysis, higher 3P CSPH probability (per 10%; SHR: 1.30, *p =* 0.039) was associated with HCC in the Vienna cohort, but no predictor remained significant after multivariable adjustment for age, sex, LSM and achievement of etiological cure (Table S5).

#### Risk stratification by Vienna 3P/5P models and other NITs compared to HVPG

**Fig. 2:**
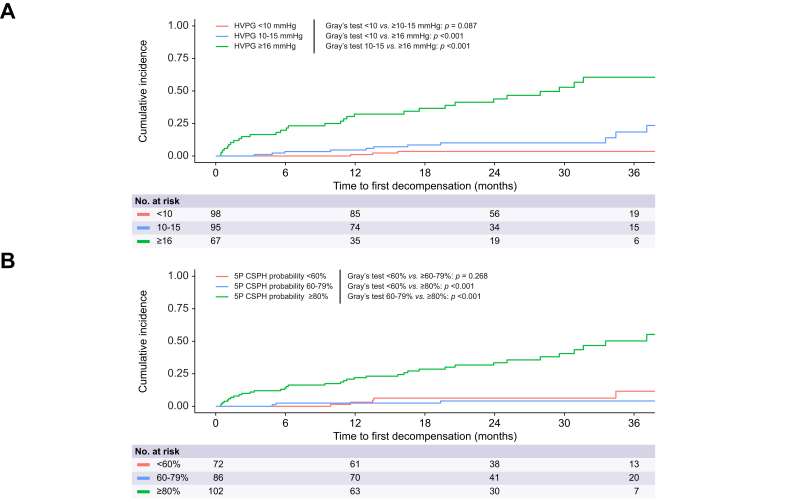
Cumulative incidence plots for first hepatic decompensation as stratified by HVPG and 5P CSPH probability in the Vienna outcome prediction cohort. Cumulative incidence of first hepatic decompensation with hepatocellular carcinoma or non-liver-related death treated as competing events as stratified by HVPG (A) and 5P CSPH Probability (B). The cumulative incidences were compared using Gray’s test. Levels of significance: *p* <0.05. CSPH, clinically significant portal hypertension; HVPG, hepatic venous pressure gradient.

During a 1-year follow-up, the cumulative incidence of first hepatic decompensation accounting for competing events was <1.1% in HVPG <10, 4.7% in HVPG 10-15 and 32.3% in HVPG ≥16 (Fig. 2). One patient without CSPH (HVPG = 6 mmHg) decompensated within a year of follow-up. This patient had an LSM of 11.3 kPa but experienced episodes of alcohol-related steatohepatitis after baseline, ultimately succumbing to acute-on-chronic liver failure after 12.5 months of follow-up. As shown in Table 3, 91.6% and 93.1% of hepatic decompensations at 2 and 3 years of follow-up, respectively, occurred in patients with CSPH.

**Table 3:** First hepatic decompensation at 1, 2 and 3 years in the Vienna outcome prediction, as stratified by different risk prediction thresholds.

Hepatic decompensation	n	Events within 1 year	Incidence proportion at 1 year	% of overall events at 1 year	Events within 2 years	Incidence proportion at 2 years	% of overall events at 2 years	Events within 3 years	Incidence proportion at 3 years	% of overall events at 3 years
HVPG										
<10 mmHg	98	1	1.0	**4**	3	3.1	**8.3**	3	3.1	**7.0**
10-15 mmHg	95	4	4.2	**16**	8	8.4	**22.2**	10	10.5	**23.3**
≥16 mmHg	67	20	29.9	**80**	25	37.3	**69.4**	30	44.8	**69.8**
LSM										
<25 kPa	145	10	6.9	**40**	15	10.3	**41.7**	19	13.1	**45.2**
≥25 kPa	106	15	14.2	**60**	21	19.8	**58.3**	23	21.7	**54.8**
ANTICIPATE±NASH CSPH probability										
<60%	106	6	5.7	**24**	8	7.5	**22.2**	10	9.4	**23.8**
≥60%	143	19	13.3	**76**	28	19.6	**77.8**	32	22.4	**76.2**
3P CSPH probability										
<60%	56	4	7.1	**16**	6	10.7	**16.7**	6	10.7	**14.0**
60-79%	99	2	2.0	**8**	3	3.0	**8.3**	6	6.1	**14.0**
≥80%	105	19	18.1	**76**	27	25.7	**75.0**	31	29.5	**72.1**
3P predicted HVPG										
<10 mmHg	18	2	11.1	**8**	4	22.2	**11.1**	4	22.2	**9.3**
10-14mmHg	176	7	4.0	**28**	11	6.3	**30.6**	15	8.5	**34.9**
≥15 mmHg	66	16	24.2	**64**	21	31.8	**58.3**	24	36.4	**55.8**
5P CSPH probability										
<60%	72	2	2.8	**8**	4	5.6	**11.1**	5	6.9	**11.6**
60-79%	86	2	2.3	**8**	3	3.5	**8.3**	3	3.5	**7.0**
≥80%	102	21	20.6	**84**	29	28.4	**80.6**	35	34.3	**81.4**
5P predicted HVPG										
<10 mmHg	31	2	6.5	**8**	4	12.9	**11.1**	4	12.9	**9.3**
10-14 mmHg	152	6	3.9	**24**	8	5.3	**22.2**	11	7.2	**25.6**
≥15 mmHg	77	17	22.1	**68**	24	31.2	**66.7**	28	36.4	**65.1**
Baveno VII CSPH rule-in criteria										
CSPH ruled out	24	1	4.2	**4**	1	4.2	**2.8**	1	4.2	**2.3**
Grey zone	130	9	6.9	**36**	14	10.8	**38.9**	19	14.6	**44.2**
CSPH ruled in	106	15	14.2	**60**	21	19.8	**58.3**	23	21.7	**53.5**

Numbers, incidence proportions and percentages of first hepatic decompensation observed in the respective strata. For nine patients reliable LSM data was not available. For 11 patients reliable data for calculation of the ANTICIPATE±NASH CSPH probability (LSM and/or platelet count) was missing. CSPH, clinically significant portal hypertension; HVPG, hepatic venous pressure gradient; LSM, liver stiffness measurement.

Notably, 21 out of 25 patients who decompensated in the first year had a 5P CSPH probability of ≥80%. Similarly, 17 out of 25 decompensations at 1 year occurred in patients with a 5P predicted HVPG of ≥15 mmHg while only two decompensations occurred in the 5P models’ respective lower-risk groups.

The Vienna 3P model slightly underperforms compared to the 5P model, while showing similar trends. At 1-year follow-up, 76% of decompensations occurred in patients with a CSPH probability ≥80%, compared to 64% in the ≥15 mmHg 3P predicted HVPG stratum. However, the incidence proportion in the latter group was the second highest after HVPG (24.2% *vs.* 29.9%).

Most patients who decompensated within the first year of follow-up had an ANTICIPATE±NASH CSPH probability ≥60% (n = 19/25), though stratification into the high-risk group yielded a low incidence proportion of 13.3%. Using only LSM, 15 of the 25 decompensating patients were correctly identified.

**Fig. 3:**
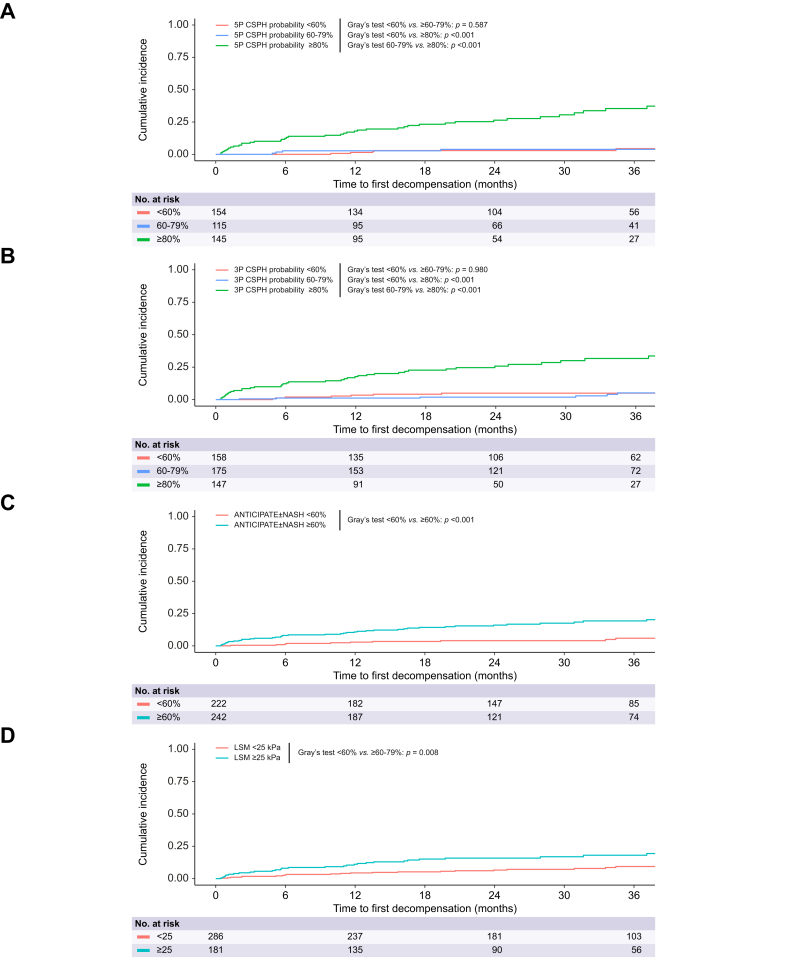
Cumulative incidence plots for first hepatic decompensation as stratified by proposed decision rules to identify patients at high risk of decompensation in the combined Vienna and Hannover cohorts. Cumulative incidence of first hepatic decompensation with hepatocellular carcinoma or non-liver-related death treated as competing events as stratified by 5P CSPH probability (A), 3P CSPH probability (B), ANTICIPATE±NASH CSPH probability (C) and LSM (D). The cumulative incidences were compared using Gray’s test. Levels of significance: *p <*0.05. CSPH, clinically significant portal hypertension; HVPG, hepatic venous pressure gradient; LSM, liver stiffness measurement.

Each decision rule demonstrated significant differentiation between a lower and higher risk patient group as assessed by Grays’s test (Fig. 3 and Fig. S7). This was also observed for the 3P and 5P models in both patients who achieved etiological cure and those who did not (cured: all Gray’s test *p* ≤0.005; not cured: all *p <*0.001; Fig. S9).

Further, risk stratification analysis in the outcome prediction CTP A sub-cohort showed similar results, except for the 3P predicted HVPG, which showed only moderate stratification ability due to a disproportionately high number of patients assigned to the medium-risk group (Fig. S8). Moreover, in this CTP A-only cohort, the ANTICIPATE±NASH model and LSM no longer provided significant discrimination between low- and high-risk groups.

### HVPG prediction cohort

#### Diagnostic performance of Vienna 3P/5P-, ANTICIPATE±NASH models and LSM

All models correlated significantly with HVPG (*p <*0.001 for all, Fig. S3) and yielded higher values in patients with CSPH (*p <*0.001 for all, Fig. S4). The ANTICIPATE±NASH model showed the highest AUROC for CSPH prediction (0.837), followed by LSM (0.807) and the Vienna 5P (CSPH probability: 0.704) and 3P (CSPH probability: 0.672) models. For severe PH, the 5P and 3P CSPH probabilities achieved AUROCs of 0.800 and 0.750, respectively (Fig. S2). Calibration was satisfactory for the 3P/5P models (Fig. S5).

The complete set of results is provided in the supplementary information.

#### Hannover cohort

Over a median follow-up of 50.3 (IQR 25.0-90.9) months, first hepatic decompensation occurred in 15 patients (7.0%), HCC developed in 24 (11.2%), 4 (1.8%) underwent liver transplantation and 13 (6.0%) died.

Time-dependent AUROCs for predicting first hepatic decompensation within 24 months ranged from 0.773-0.814 for the 5P and 0.792-0.818 for the 3P model (Fig. 3). Both models out-performed LSM at every assessed time-point (all *p <*0.05). From 12 months onward, 3P also showed significantly higher discrimination than ANTICIPATE±NASH (all *p <*0.05). In adjusted Fine and Gray competing risk regression modelling, the 5P model emerged as an independent risk factor for incident decompensation (CSPH probability: aSHR 2.20, *p <*0.001; Table 2). Concordantly, applying the same decision rules as in the Vienna cohorts, differentiation into risk groups also proved significant for the 3P (CSPH probability: <60% *vs.* ≥80% Gray’s test *p <*0.001, 60-79% *vs.* ≥80% *p =* 0.013, <60% *vs.* 60-79% *p =* 0.548) and 5P (CSPH probability: <60% *vs*. ≥80% *p =* 0.001, 60-79% *vs.* ≥80% *p =* 0.370, <60% *vs.* 60-79% *p =* 0.046) models in the Hannover cohort.

Furthermore, the 3P model (predicted HVPG; aSHR: 1.48, *p =* 0.027) was an independent predictor of liver-related death. For HCC occurrence, the 3P and 5P models, LSM and the ANTICIPATE±NASH model were significantly associated in univariable analysis (all *p* ≤0.01). In multivariable analysis, only 5P CSPH probability (*p =* 0.034) remained statistically significant, while the 3P model and the ANTICIPATE±NASH model showed borderline associations (Table S5).

## Discussion

NITs have transformed the diagnosis and management of patients with cACLD at risk for CSPH, with the paradigm-shifting towards initiating treatment with NSBBs as soon as CSPH is detected by non-invasive measures.^2^^,^^34^

In our study of 266 HVPG-characterized patients with cACLD – representing a contemporary cohort with diverse etiologies, including 17.7% with MASLD – we aimed to validate the diagnostic and prognostic value of the recently developed Vienna 3P/5P MLMs.^20^ These models were trained on patients with various etiologies of cACLD and use only standard laboratory parameters, aiming to assist clinicians to identify patients at risk of first hepatic decompensation without the need for invasive hepatic vein catheterization or non-blood-based NITs such as LSM.^20^ We compared their prognostic performance to HVPG and other widely used NITs in a large Viennese and an external validation cohort provided by the Medical School Hannover.

Comparison of the models’ outputs – CSPH probability and predicted HVPG – reveals that using CSPH probability provides more reliable calibration for CSPH detection (Fig. S5). Consequently, emphasis is placed on the CSPH probability as the more robust 3P/5P model output measure, with the respective model’s calculated CSPH probabilities referenced whenever a model is mentioned in the following discussion.

Our results show that the Vienna 3P/5P models significantly correlate with HVPG. The Vienna 5P model demonstrated adequate discrimination and accurate calibration, achieving an AUROC of 0.704 for predicting CSPH. We observed higher AUROCs for CSPH prediction with the ANTICIPATE±NASH model (0.837) and LSM (0.807), while the 3P/5P models performed well for severe PH (*i.e*. HVPG ≥16 mmHg) discrimination, achieving AUROCs of 0.800 (5P) and 0.750 (3P). However, a key advantage of the 3P/5P models is that they – unlike the ANTICIPATE±NASH model and LSM, which require elastography, lab ± clinical assessments – rely solely on blood parameters.

When evaluating prognostic capabilities, the choice of prediction horizon is determined by clinical context. Blood-based models such as the 3P/5P are easily repeatable and thus allow for frequent risk reclassification. This makes short-term prediction valuable for identifying patients at high near-term risk who may benefit from closer monitoring or timely optimization of modifiable factors. At the same time, because models like ANTICIPATE±NASH are recommended in Baveno VII^2^ for CSPH identification and possible NSBB initiation, we also report longer-term risk estimates for the 3P/5P models. These are particularly relevant given that NSBB therapy reduces the risk of decompensation over the long term.^8^

Firstly, we confirmed HVPG as an independent predictor of first hepatic decompensation in multivariable competing risk regression analysis. Consistent with this, HVPG also achieved high time-dependent AUROCs for first hepatic decompensation across 3 years of follow-up.

Next, we found that the 3P and 5P models predicted first hepatic decompensation in multivariable analysis independent from age, sex, LSM, achievement of etiological cure and NSBB intake (with 73.1% of patients with CSPH being on NSBB therapy). The 5P model further showed comparable prediction capabilities to HVPG with similar time-dependent AUROCS for first hepatic decompensation throughout a 3-year follow-up period. Notably, for short-term decompensation within 6 months, the 5P model outperformed the ANTICIPATE±NASH model and LSM. These findings were also validated in the external Hannover cohort, where LSM was significantly outperformed by both the 3P and 5P models, while the 3P model was also superior to the ANTICIPATE±NASH model from the 12-month timepoint onward (Fig. 1). The 5P model also remained an independent risk factor in adjusted multivariable Fine and Gray modelling in the Hannover cohort.

A more detailed look into the Vienna cohort revealed that risk-based stratification of patients by 5P CSPH probability is highly efficient, as 21 (84%) decompensations occurred in the “high-risk” group, while only 2 (8%) decompensations were observed in its “lower-risk” group in the first year of follow-up – comparable to stratification by HVPG with 20 (80%) events in HVPG ≥16 mmHg und 1 (4%) in <10 mmHg. Importantly, these results were achieved with relatively balanced allocation of patients across risk groups. 5P’s stratification proved superior to the ANTICIPATE±NASH model and LSM, as the latter two showed higher numbers of decompensations in their respective lower-risk groups and fewer in their high-risk groups (Table 3). Additionally, 5P CSPH probability provided more appropriate incidence proportions for decompensation. Also, the 3P model worked well in detecting patients at high risk for decompensation, identifying 19 out of 25 (76%) in the first year of follow-up, which corresponds to the performance of the ANTICIPATE±NASH model. When compared to the Baveno VII suggested decision rules, the 3P/5P models again appeared superior, as relatively high numbers of decompensations were observed among patients in the diagnostic grey zone of the Baveno VII criteria. Moreover, sensitivity analysis in the outcome prediction CTP A sub-cohort demonstrated that the 3P/5P models retained their ability to significantly distinguish between higher and lower-risk groups in CTP-A-only patients. In contrast, this discrimination was no longer observed for the ANTICIPATE±NASH model and LSM in this patient group.

The superior “short-term” prognostic performance of the 5P model compared to ANTICIPATE±NASH and LSM may also be attributed to its high AUROC for detecting severe PH, as most decompensation events occurred in patients with an HVPG ≥16 mmHg – an important HVPG risk cut-off that has recently been validated for the specific etiology of MASLD.^4^ Notably, the ANTICIPATE±NASH model and LSM were both significant predictors of hepatic decompensation in our cohort.

This indicates that accurate prediction of a binary outcome, such as CSPH, may appropriately identify patients at risk but not necessarily fully capture absolute decompensation risk, given the considerable variability within the CSPH classification itself. For instance, a patient with an HVPG of 11 mmHg has a markedly different risk profile compared to one with an HVPG of 25 mmHg.

With regards to NSBB therapy, there has been an ongoing debate on when to initiate treatment. A re-analysis of the PREDESCI data recently suggested a substantial population-level benefit in terms of decompensation-free survival when NSBBs are initiated upon CSPH diagnosis.^35^

In line with this, we assessed the potential effectiveness of hypothetical NSBB treatment initiation within our cohort. Using HVPG-diagnosed CSPH as the criterion, 162 patients would receive NSBB treatment per current consensus guidelines,^2^ with 24 of these patients experiencing decompensation within 1 year. Alternatively, if NSBBs were initiated in patients with a 5P CSPH probability ≥80%, only 102 patients would be treated, with 21 decompensations being observed in this group. Expanding treatment to include patients in the medium-risk group (5P CSPH probability of 60-79%) would result in 188 patients receiving treatment, with 23 experiencing decompensation within a year of follow-up.

Based on these observations, the 5P model effectively identifies patients at a comparable baseline risk as CSPH, while also proficiently detecting patients at high risk for decompensation, implying sufficient capability to select patients who would benefit from NSBB therapy.

However, the recommendation to initiate NSBB treatment solely based on the Vienna 5P model’s results cannot be given as of today, as current consensus guidelines^2^ require diagnosis of CSPH via HVPG measurement or specified NIT-based algorithms. This is, in part, due to the fact that NSBBs have been shown to effectively reduce HVPG to a much greater extent in patients diagnosed with CSPH.^8^ As discussed earlier, while LSM and the ANTICIPATE±NASH model showed higher accuracy for CSPH, the 3P and particularly the 5P model offered stronger risk stratification.

In the context of NSBB treatment initiation, this raises the question of whether the scenario of the 5P model possibly classifying patients “only” exhibiting subclinical PH (6-9 mmHg) as high-risk, limits their applicability for NSBB treatment. However, given that PH is the primary driver of decompensation in cACLD,^36^ the observed superior risk stratification capabilities might suggest that models, such as the 5P model, may more accurately capture PH progression over the (near) future compared to predictors that are more precise in binary CSPH prediction, particularly when relying on single timepoint measurements. This might be especially relevant in patients with active/progressive cACLD and possibly arises from such models comprising markers that indicate (potentially early) liver dysfunction. However, additional studies with similar observations in external cohorts are necessary to validate this hypothesis. As of now, frequent monitoring with accurate CSPH surrogates, as practiced in many large centers, seems to provide the best insight into PH status and dynamics as well as associated risk over time,^37^^,^^38^ though such an approach may not be feasible outside major centers. Especially, but not exclusively in settings with limited resources, initial and possibly subsequent risk stratification using easily repeatable models, such as the 5P model, could serve as a practical alternative or complementary approach to enhance patient care.

In the context of the above-described scenario, NSBB therapy based on 5P stratification may still represent a clinically rational strategy for prophylaxis even in subclinical PH, especially in patients with significant comorbidities. As these patients seem likely to progress to CSPH, given the high event rate in identified risk groups, NSBB therapy could then potentially reduce HVPG, delivering clinically meaningful outcomes.

With the 3P/5P models incorporating only widely available routine laboratory parameters, they might effectively address a gap currently withholding universal applicability of blood-based risk stratification approaches for cACLD, such as the recently proposed algorithm based on FIB-4.^39^ Here, a FIB-4 cut-off ≥1.75 demonstrated predictive capacity for liver-related events comparable to an LSM threshold of 10 kPa – omitting the need for non-blood-based and costly elastographic tests. However, reliance on FIB-4 alone has been shown to only provide limited value beyond identifying advanced fibrosis.^40^

In this context, the Vienna 3P/5P models may offer added clinical value by combining robust prognostic accuracy with broad applicability, independence from specialized equipment or operator expertise, and the capacity for repeated measurements without additional patient burden, which may support individualized monitoring, especially, but not limited to, resource-limited settings. Moreover, the models’ predictive performance was maintained irrespective of etiological cure status, which is an important observation as this information is often unavailable in routine practice. Upon validation in further external cohorts, the 5P model in particular may be appropriate to identify patients who could benefit from the initiation of NSBB therapy, given its strong prognostic implications for predicting PH-driven complications.

A key limitation of our study is a potential selection bias, as HVPG measurements are typically done in patients with suspected ACLD or PH, which may lead to a higher prevalence of CSPH in our tertiary care cohort. Moreover, impaired liver function might contribute to falsely positive results achieved by the 3P/5P models as they comprise parameters reflecting liver dysfunction. To account for this, sub-cohort analysis on patients solely exhibiting CTP A was conducted, enabling us to validate our findings. Secondly, a part (48.1%) of the outcome prediction cohort included patients who were initially part of the training cohort used to develop the Vienna 3P/5P models. This might introduce a bias with potentially overfitted data, possibly affecting the generalizability of our findings. However, it is crucial to emphasize that the models were not trained (developed) on clinical outcomes. Assessment of the 3P/5P models’ capabilities of correlating to or predicting CSPH and severe PH was strictly done in the independent HVPG prediction cohort. Thirdly, the Hannover cohort used for external validation consists predominantly of patients with viral hepatitis, which limits direct etiological comparability to the contemporary and diverse Vienna cohort. Importantly, hepatitis remains a major cause of chronic liver disease worldwide and is still frequently encountered in clinical practice, despite the availability of effective antiviral therapies. We included the Hannover cohort to demonstrate the models’ performance across different etiological backgrounds and clinical settings, where the non-comparability of cohorts actually strengthens the assessment of external validity and generalizability. Fourthly, at our center most patients were/are treated with NSBBs even prior to the findings of the PREDESCI study^8^^,^^34^ – potentially lowering decompensation risk among “high-risk” patients. To account for this, we adjusted multivariable models for NSBB intake.

In conclusion, we could validate the value of the blood-based Vienna 3P/5P models for identification of patients with cACLD at risk of decompensation in an internal and external validation cohort, with the 5P model (based on bilirubin, PLT, activated partial thromboplastin time, cholinesterase and gamma-glutamyltransferase) being preferrable. This study goes beyond the previous work of our group that introduced the 3P and 5P models as non-invasive tools for CSPH risk assessment,^20^ as we could show that both models were able to predict the occurrence of decompensation with high accuracy – while not requiring non-universally available HVPG, elastography or non-routine laboratory parameters. Notably, the 5P model performed comparable to invasively measured HVPG while both, 3P and 5P risk stratification criteria effectively identified patients with a considerable short-term risk of decompensation, underscoring their value in selecting patients with cACLD who may benefit from timely therapeutic interventions intending to prevent hepatic decompensation.

## Abbreviations

(c)ACLD, (compensated) advanced chronic liver disease; aSHR, adjusted subdistribution hazard ratio; CSPH, clinically significant portal hypertension; CTP, Child-Turcotte-Pugh; FIB-4, fibrosis-4; HCC, hepatocellular carcinoma; HVPG, hepatic venous pressure gradient; LSM, liver stiffness measurement; MASLD, metabolic-dysfunction-associated steatotic liver disease; MLM, machine learning model; NIT, non-invasive test; NSBB, non-selective beta blockers; PH, portal hypertension; PLT, platelet count; SSM, spleen stiffness measurement; VCTE, vibration-controlled transient elastography; VWF, von Willebrand factor.

## Authors’ contributions

All authors contributed either to study concept and design (G.K, T.R.) and/or data acquisition (all authors), analysis (G.K., T.R.) or interpretation (all authors). G.K and T.R. drafted the manuscript, which was critically revised by all other authors. All authors read and approved the final manuscript.

## Data availability

The data that support the findings of this study are available from the corresponding author upon reasonable request.

## Financial support

No financial support specific to this study was received. Some authors (BS, BSH, PS, TR) were co-supported by the Austrian Federal Ministry for Digital and Economic Affairs, the 10.13039/100010132National Foundation for Research, Technology and Development, the 10.13039/501100006012Christian Doppler Research Association, and 10.13039/100008349Boehringer Ingelheim. Some authors (GK, BS, BSH, CS, GS, MJ, MS, LH, LB, ND, PS, PT, MT, MM, TR) were supported by the Clinical Research Group MOTION, 10.13039/501100005788Medical University of Vienna, Vienna, Austria – a project funded by the Clinical Research Groups Program of the 10.13039/501100016168Ludwig Boltzmann Gesellschaft (Grant Nr: LBG_KFG_22_32) with funds from the Fonds Zukunft Österreich.

## Conflict of interest

G.K. has nothing to disclose. B.S. received travel support from AbbVie, Gilead, and Falk. M.J. served as speaker and/or consultant for Gilead Sciences Inc. B.S.H. received travel support from Ipsen and Falk. C.S. has nothing to disclose. M.S. received travel support from MSD, Sandoz, BMS, AbbVie and Gilead; and speaking honoraria from BMS and Gilead. N.D. has nothing to disclose. L.H. has nothing to disclose. L.B. has nothing to disclose. G.S. received travel support from Amgen. P.T. has nothing to disclose. J.R. has nothing to disclose. S.G. has nothing to disclose. B.M. received speaker and/or consulting fees from Abbott Molecular, Astellas, Intercept, Falk, AbbVie, Luvos, Norgine, Gore, Gilead, Fujirebio, Merck (MSD), and Roche. He also received research support from Abbott Molecular, Altona Diagnostics, EWIMED, Fujirebio and Roche. E.A. has nothing to disclose. M.S.M. has nothing to disclose. O.P. has nothing to disclose. P.S. received consulting fees from PharmaIN and travel support from Falk Pharma. A.S. served as a speaker and/or consultant and/or advisory board member for Boehringer Ingelheim, Gilead, and MSD. M.T. received speaker fees from Agomab, BMS, Chemomab, Falk Foundation, Gilead, Intercept, Ipsen, Jannsen, Madrigal, MSD, and Roche; advised for AbbVie, Albireo, BiomX, Boehringer Ingelheim, Cymabay, Falk Pharma GmbH, Genfit, Gilead, Hightide, Intercept, Ipsen, Janssen, MSD, Novartis, Phenex, Pliant, Rectify, Regulus, Siemens, and Shire; received travel support from AbbVie, Falk, Gilead, Intercept, and Jannsen; received research grants from Albireo, Alnylam, Cymabay, Falk, Gilead, Intercept, MSD, Takeda, and UltraGenyx; and is also a co-inventor of patents on the medical use of norUDCA filed by the Medical Universities of Graz and Vienna. M.M. served as a speaker and/or consultant and/or advisory board member for AbbVie, Echosens, Eli Lilly, Falk, Gilead, Ipsen, Takeda, and W. L. Gore & Associates; received travel support from AbbVie and Gilead; and received grants/research support from Echosens. T.R. received grant support from Abbvie, Boehringer Ingelheim, Gilead, Intercept/Advanz Pharma, MSD, Myr Pharmaceuticals, Philips Healthcare, Pliant, Siemens, and W. L. Gore & Associates; speaking honoraria from Abbvie, Echosens, Gilead, Intercept/Advanz Pharma, Roche, MSD, and W. L. Gore & Associates; consulting/advisory board fees from Abbvie, Astra Zeneca, Bayer, Boehringer Ingelheim, Gilead, Intercept/Advanz Pharma, MSD, Resolution Therapeutics, and Siemens; and travel support from Abbvie, Boehringer Ingelheim, Dr. Falk Pharma, Gilead and Roche.

Please refer to the accompanying ICMJE disclosure forms for further details.
