# Supplementary material for: Blood-based Vienna 3P/5P risk models accurately predict first hepatic decompensation in compensated advanced chronic liver disease
Source: JHEP Rep. 2025 Oct 17;8(2):101642. doi: 10.1016/j.jhepr.2025.101642 (PMC12810536; doi:10.1016/j.jhepr.2025.101642)
Supplement: Multimedia component 1 [file mmc1.pdf]

Blood-based Vienna 3P/5P risk models accurately predict first hepatic decompensation in compensated advanced chronic liver disease☆

Georg Kramer, Benedikt Simbrunner, Mathias Jachs, Lorenz Balcar, Benedikt Silvester Hofer, Nina Dominik, Lukas Hartl, Michael Schwarz, Georg Semmler, Christian Sebesta, Paul Thöne, Sophia Geisselbrecht, Benjamin Maasoumy, Eduardo Alvarez, Martin Sebastian McCoy, Oleksandr Petrenko, Jiří Reiniš, Philipp Schwabl, Albert F. Stättermayer, Michael Trauner, Mattias Mandorfer, Thomas Reiberger

Table of contents

Supplementary results…………………………………………………………………....2

Supplementary tables……………………………………………………………………3

Supplementary figures…………………………………………………………………..11

Supplementary results

Incidence of Liver transplantation.

During a median follow-up of 23.9 months (IQR 15.3–32.6) in the Vienna Outcome Prediction cohort, liver transplantation (LT) was performed in 11 patients (4.2%). In most cases (n=7), hepatic decompensation preceded LT, with transplantation subsequently undertaken due to disease progression. Two additional patients underwent LT for hepatocellular carcinoma (HCC). Of the remaining two patients who reached LT without preceding hepatic decompensation or HCC, both had progressive cholestatic cirrhosis (primary sclerosing cholangitis or primary biliary cholangitis) and were transplanted shortly after listing and did not develop decompensation (ascites, hepatic encephalopathy, or variceal bleeding) before LT. In the Hannover cohort (median follow-up 50.3 months, IQR 25.0-90.9), all four LTs (1.8%) were performed for HCC.

Vienna HVPG prediction cohort

Diagnostic Accuracy of Vienna 3P/5P and ANTICIPATE±NASH Models for CSPH and Severe PH.

The ANTICIPATE±NASH model showed the highest AUROC for predicting clinically significant portal hypertension (CSPH) (0.837 [95%CI: 0.766-0.907]) followed by liver stiffness measurement (LSM) (0.807 [0.732-0.882]), the Vienna 5P model (CSPH probability (0.704 [0.613-0.796]), predicted HVPG (0.699 [0.608-0.789])) and 3P model (CSPH probability (0.672 [0.579-0.765]), predicted HVPG (0.680 [0.587-0.772])). For the detection of severe PH, the 5P CSPH Probability yielded an AUROC of 0.800 (0.711-0.889) while the 3P CSPH probability achieved an AUROC of 0.750 (0.642-0.858) (Fig. S2; Table S2). The 3P/5P models' severe PH probabilities achieved comparable AUROCs (Fig. S10) and were subsequently not further assessed. AUROCs calculated within the HVPG prediction CTP A Sub-Cohort were also similar to the overall HVPG prediction cohort, as presented in Table S8.

Overall calibration for CSPH was satisfactory, as reflected by acceptable Brier scores for both the 5P model (CSPH probability: 0.219; predicted HVPG: 0.214) and the 3P model (CSPH probability: 0.231; predicted HVPG: 0.230) (Fig. S5).

Thresholds for ruling in ruling in or ruling out CSPH.

As per Youden's Index, the Vienna 5P model showed an optimal CSPH probability threshold of 74.6% and predicted HVPG of 14.8 mmHg, while the 3P model yielded thresholds of 86.2% for CSPH probability and 14.9 mmHg for predicted HVPG. For the sake of completeness, calculations were also done for the ANTICIPATE±NASH model, yielding an optimal threshold of 66.58%, and for LSM, achieving a threshold of 25.65 kPa, aligning well with established cutoffs.

Supplementary tables

Table S1. Patient characteristics of the Vienna cohorts.

	HVPG prediction cohort	Outcome prediction cohort	*p*-value
**Participants, n**	131	260	
**Age, years (median, IQR)**	56.7 (48.8-63.55)	57.3 (48.75-64.15)	0.692
**Sex, n (%)**			0.929
Male	88 (67.2%)	172 (66.2%)	
Female	43 (32.8%)	88 (33.8%)	
**BMI, kg/m2 (IQR)**	26.59 (23.35-30.56)	26.845 (23.44-30.58)	0.951
BMI ≥ 30, n (%)	40 (30.53 %)	79 (30.38 %)	
**Diabetes, n (%)**	28 (21.4%)	57 (21.9%)	0.921
**Etiology, n (%)**			0.868
ALD	33 (25.2%)	61 (23.5%)	0.753
Viral hepatitis	30 (22.9%)	72 (27.7%)	0.403
ALD + viral hepatitis	10 (7.6%)	14 (5.4%)	0.496
MASLD	23 (17.6%)	47 (18.1%)	0.999
Cholestatic	10 (7.6%)	23 (8.8%)	0.855
Other	22 (16.8%)	40 (15.4%)	0.794
**HVPG, median (IQR)**	10 (7-13.5)	11 (7.88-16)	**0.032**
CSPH, n (%)	74 (56.5%)	162 (62.3%)	0.317
Severe PH, n (%)	21 (16%)	67 (25.8%)	**0.041**
**Varices**			
Small, n (%)	29 (22.1%)	67 (25.8%)	0.715
Large, n (%)	7 (5.3%)	27 (10.4%)	0.183
**Baveno VII CSPH criteria**			0.879
CSPH ruled-in, n (%)	54 (41.2%)	106 (40.8%)	0.999
Grey zone, n (%)	63 (48.1%)	130 (50%)	0.803
CSPH ruled-out, n (%)	14 (10.7%)	24 (9.2%)	0.781
**Child-Pugh Stage**			0.096
A	101 (77.1%)	219 (84.2%)	0.112
B	30 (22.9%)	41 (15.8%)	0.112
C	0 (0%)	0 (0%)	-
**VCTE-LSM, kPa median (IQR)**	22.3 (15.6-39.6)	22.3 (15.5-36.3)	0.976
**ANTICIPATE±NASH CSPH Probability (%)**	69.74 (58.08-87.77)	71.85 (38.69-92.71)	0.702
**Vienna 3P Model**			
CSPH Probability (%)	78.82 (63.20-89.65)	76.04 (62.96-86.72)	0.367
Predicted HVPG, mmHg (IQR)	13.9 (12.23-15.35)	13.77 (12.32-15.06)	0.585
**Vienna 5P Model**			
CSPH Probability (%)	74.62 (58.08-87.77)	74.68 (57.95-87.12)	0.917
Predicted HVPG, mmHg (IQR)	13.59 (11.59-15.77)	13.4 (11.53-15.59)	0.845
**MELD, median (IQR)**	10 (8-13)	9 (8-12)	0.129
**Platelet count, G/L, median (IQR)**	117 (84.5-171)	110 (83-160)	0.353
**Bilirubin, mg/dl, median (IQR)**	0.94 (0.61-1.925)	0.915 (0.6175-1.52)	0.289
**INR, median (IQR)**	1.3 (1.1-1.4)	1.2 (1.1-1.4)	0.330
**aPTT, s, median (IQR)**	38.3 (35.7-41.85)	38.1 (35.2-41.425)	0.444
**Albumin, g/L, median (IQR)**	38.7 (36.05-42.05)	39.1 (36.1-41.725)	0.941
**CHE, kU/L, median (IQR)**	5.31 (4.08-6.645)	5.42 (4.085-6.985)	0.479
**GGT, U/L, median (IQR)**	85 (43.5-191)	96 (47.75-189.5)	0.677

Data expressed as n (%) or median (IQR). Continuous variables were analyzed using either an Independent Samples t-test or Mann-Whitney U test, depending on whether the data followed a normal distribution. Categorical variables were compared using Pearson's Chi-squared test or Fisher's exact test. Abbreviations: ALD, Alcohol-related liver disease; aPTT, activated partial thromboplastin time; BMI, Body-Mass-Index; CHE, Cholinesterase; CSPH, Clinically significant portal hypertension; GGT, Gamma-glutamyl Transferase; HVPG, hepatic venous pressure gradient; INR, international normalized ratio; MASLD, Metabolic dysfunction- associated steatotic liver disease; MELD, model for end-stage liver disease; PH, portal hypertension; VCTE-LSM, vibration-controlled transient elastography liver stiffness measurement. *P*-values in bold indicate statistical significance.

Table S2. DeLong Tests' results comparing AUROCs for predicting CSPH in the Vienna HVPG prediction Cohort.

	**LSM**	**3P CSPH Probability**	**5P CSPH Probability**	**3P Predicted HVPG**	**5P Predicted HVPG**	**ANTICIPATE±NASH CSPH Probability**
**LSM**	-	**0.01**	0.057	**0.017**	**0.034**	0.093
**3P CSPH Probability**	-	-	0.503	0.499	0.458	**<0.001**
**5P CSPH Probability**	-	-	-	0.613	0.803	**0.007**
**3P Predicted HVPG**	-	-	-	-	0.599	**<0.001**
**5P Predicted HVPG**	-	-	-	-	-	**0.003**
**ANTICIPATE±NASH CSPH Probability**	-	-	-	-	-	-

DeLong test's results of the comparison of each predictor's AUROC for prediction of CSPH are presented as *p* values. Abbreviations: AUROC, area under the receiver operating characteristic curve; CSPH, clinically significant portal hypertension; HVPG, hepatic venous pressure gradient; PH, portal hypertension. *P*-values in bold indicate statistical significance.

Table S3. DeLong Tests' results comparing AUROCs for predicting severe PH in the HVPG prediction Cohort.

	5P CSPH Probability	3P Predicted HVPG	5P Predicted HVPG
**3P CSPH Probability**	0.219	0.836	0.152
**5P CSPH Probability**	-	0.208	0.734
**3P Predicted HVPG**	-	-	0.140
**5P Predicted HVPG**	-	-	-

DeLong test's results of the comparison of each predictor's AUROC for prediction of severe PH are presented as *p* values. Abbreviations: AUROC, area under the receiver operating characteristic curve; CSPH, clinically significant portal hypertension; HVPG, hepatic venous pressure gradient; PH, portal hypertension. *P*-values in bold indicate statistical significance.

Table S4. Risk factors for Liver-related Mortality.

	Univariate Competing Risk Regression Model			Multivariate Competing Risk Regression Models (adjusted for Age, Sex, LSM and achievement of etiological cure) *		
	**SHR**	**95% CI**	***p*-value**	**aSHR**	**95% CI**	***p*-value**
**VIENNA**						
**Age, per year**	1.03	0.98-1.07	0.230	-	-	-
**Sex, male**	2.08	0.61-7.04	0.240	-	-	-
**LSM, per kPa**	1.02	0.99-1.04	0.290	-	-	-
**MELD, per point**	1.12	1.02-1.22	**0.017**	-	-	-
**Albumin, per g/dL**	0.97	0.87-1.07	0.500	-	-	-
**Achievement of etiological cure, binary**	0.57	0.20-1.60	0.280	-	-	-
**HVPG, per mmHg**	1.11	1.02-1.20	**0.015**	1.15	1.03-1.28	**0.011**
**3P CSPH Probability, per 10%**	1.15	0.74-1.80	0.530	1.08	0.73- 1.59	0.700
**5P CSPH Probability, per 10%**	0.98	0.70-1.36	0.880	0.89	0.63- 1.24	0.480
**3P Predicted HVPG, per mmHg**	1.06	0.92-1.23	0.400	1.05	0.89-1.24	0.550
**5P Predicted HVPG, per mmHg**	1.09	0.85-1.39	0.520	1.04	0.79- 1.36	0.930
**Anticipate±NASH CSPH Probability, per 10% ***	1.01	0.84-1.21	0.920	1.02	0.86- 1.20	0.840
**HANNOVER**						
**Age, per year**	1.12	1.04-1.21	**0.002**	-	-	-
**Sex, male**	0.33	0.09-1.28	0.110	-	-	-
**LSM, per kPa**	0.99	0.95-1.05	0.880	-	-	-
**MELD, per point**	1.15	1.00-1.33	**0.048**	-	-	-
**Albumin, per g/dL**	0.93	0.87-0.99	**0.035**	-	-	-
**Achievement of etiological cure, binary**	0.03	0.01-0.25	**0.002**	-	-	-
**3P CSPH Probability, per 10%**	1.43	0.94-2.18	0.100	1.43	0.97-2.11	0.073
**5P CSPH Probability, per 10%**	1.19	0.89-1.60	0.250	1.30	0.90-1.88	0.160
**3P Predicted HVPG, per mmHg**	1.71	0.94-1.72	0.120	1.48	1.05-2.09	**0.027**
**5P Predicted HVPG, per mmHg**	1.09	0.93-1.28	0.270	1.13	0.90-1.41	0.280
**Anticipate±NASH CSPH Probability, per 10% ***	1.01	0.82-1.24	0.920	1.09	0.81-1.46	0.570

Uni- and multivariate competing risk regression models assessing predictors for liver-related mortality. The multivariate models incorporate either HVPG, the Vienna 3P or 5P model or the ANTICIPATE±NASH model. Liver Transplantation and non-liver-related death were treated as competing events. The results are presented as SHRs or aSHRs, along with 95% CIs and *p*-values. *P*-values in bold indicate statistical significance.

* The multivariate model incorporating the ANTICIPATE±NASH CSPH Probability was adjusted to age, sex, achievement of etiological cure and albumin to avoid overfitting.

Abbreviations: aSHR, adjusted SHR; CRP, C-reactive protein; CSPH, clinically significant portal hypertension; HVPG, hepatic venous pressure gradient; LSM, liver stiffness measurement; MELD: model for end-stage liver disease; SHR, subdistribution hazard ratio.

Table S5. Risk factors for Hepatocellular Carcinoma.

	**Univariate Competing Risk Regression Model**			**Multivariate Competing Risk Regression Models (adjusted for Age, Sex, LSM and achievement of etiological cure) ***		
	**SHR**	**95% CI**	***p*-value**	**aSHR**	**95% CI**	***p* value|**
**VIENNA**						
**Age, per year**	1.02	0.97-1.07	0.460	-	-	-
**Sex, male**	2.01	0.57-7.11	0.280	-	-	-
**LSM, per kPa**	1.01	0.98-1.04	0.510	-	-	-
**MELD, per point**	1.06	0.98-1.16	0.170	-	-	-
**Albumin, per g/dL**	0.99	0.97-1.16	0.880	-	-	-
**Achievement of etiological cure, binary**	2.50	0.85-7.39	0.097	-	-	-
**HVPG, per mmHg**	1.07	0.98-1.12	0.150	1.05	0.94- 1.18	0.360
**3P CSPH Probability, per 10%**	1.30	1.01-1.68	**0.039**	1.24	0.78- 1.95	0.370
**5P CSPH Probability, per 10%**	1.11	0.90-1.36	0.330	1.02	0.78- 1.33	0.910
**3P Predicted HVPG, per mmHg**	1.04	0.95-1.13	0.380	1.00	0.81- 1.23	0.999
**5P Predicted HVPG, per mmHg**	1.06	0.93-1.21	0.350	0.99	0.80- 1.23	0.940
**Anticipate±NASH CSPH Probability, per 10% ***	1.01	0.84-1.21	0.940	0.99	0.82- 1.19	0.920
**HANNOVER**						
**Age, per year**	1.01	0.97-1.04	0.700	-	-	-
**Sex, male**	8.36	1.92-36.3	**0.005**	-	-	-
**LSM, per kPa**	1.03	1.01-1.05	**0.006**	-	-	-
**MELD, per point**	1.15	1.04-1.28	**0.007**	-	-	-
**Albumin, per g/dL**	0.91	0.85-0.97	**0.002**	-	-	-
**3P CSPH Probability, per 10%**	1.48	1.12-1.95	**0.006**	1.31	0.97-1.77	0.082
**5P CSPH Probability, per 10%**	1.43	1.12-1.82	**0.004**	1.30	1.02-1.67	**0.034**
**3P Predicted HVPG, per mmHg**	1.38	1.11-1.70	**0.003**	1.25	0.99-1.58	0.057
**5P Predicted HVPG, per mmHg**	1.16	1.06-1.27	**0.001**	1.09	0.99-1.20	0.088
**Anticipate±NASH CSPH Probability, per 10% ***	1.30	1.09-1.55	**0.003**	1.17	0.99-1.39	0.067

Uni- and multivariate competing risk regression models assessing predictors for Hepatocellular Carcinoma. The multivariate models presented incorporate either HVPG, the Vienna 3P/5P models or the ANTICIPATE±NASH model. Liver Transplantation and all-cause death were considered as competing events. The results are presented as SHRs or aSHRs, along with 95% CIs and *p*-values. *P*-values in bold indicate statistical significance.

* The multivariate model incorporating the ANTICIPATE±NASH CSPH Probability was adjusted to age, sex, achievement of etiological cure and albumin to avoid overfitting. In the Hannover cohort, 'achievement of etiological cure' was omitted as an adjustment variable because every event of interest occurred in patients who had already achieved cure, leaving no variability to model.

Abbreviations: aSHR, adjusted SHR; CSPH, clinically significant portal hypertension; HVPG, hepatic venous pressure gradient; LSM, liver stiffness measurement; SHR, subdistribution hazard ratio.

Table S6. Time-dependent AUROCs for predicting first hepatic decompensation.

	6 months	12 months	18 months	24 months	36 months
**VIENNA**					
**HVPG**	0.822	0.822	0.783	0.804	0.811
**3P CSPH Probability**	0.742	0.733*	0.731	0.703	0.699
**3P predicted HVPG**	0.752	0.732*	0.734	0.700*	0.703
**5P CSPH Probability**	0.815	0.753	0.735	0.728	0.787
**5P predicted HVPG**	0.801	0.758	0.743	0.738	0.782
**LSM**	0.621*†	0.636**	0.677	0.68*	0.616**†
**ANTICIPATE±NASH CSPH Probability**	0.689*†	0.691**	0.716	0.703*	0.687*
**HANNOVER**					
**3P CSPH Probability**	0.813	0.795	0.795	0.793	0.770
**3P predicted HVPG**	0.818	0.792	0.792	0.794	0.761
**5P CSPH Probability**	0.787	0.773	0.773	0.776	0.640
**5P predicted HVPG**	0.814	0.785	0.785	0.785	0.677
**LSM**	0.748†‡	0.689†‡	0.689†‡	0.669†‡	0.576†‡
**ANTICIPATE±NASH CSPH Probability**	0.842‡	0.751‡	0.751‡	0.755‡	0.643‡

Time-dependent AUROCs for predicting first hepatic decompensation. Occurrence of hepatocellular carcinoma and non-liver related death were treated as competing events. AUROCs of HVPG, ANTICIPATE±NASH CSPH Probability and LSM were compared to the AUROCs of every other predictor by usage of the 'timeROC'-package. "*" indicate a *p* value <0.05 and "**" a *p* value <0.005 as compared to HVPG's AUROC at the specified timepoint. "†" indicates a *p*-value <0.05 as compared to one or both 5P Model's AUROCs, whereas "‡" indicates a *p*-value <0.05 as compared to one or both 3P Model's AUROCs at the specified timepoint.

Abbreviations: AUC, area under the curve; AUROC, area under the receiver operating characteristic curve; CSPH, clinically significant portal hypertension; HVPG, hepatic venous pressure gradient; LSM, liver stiffness measurement; PH, portal hypertension, ROC, receiver operating characteristic.

Table S7. AUROCs for prediction of CSPH and severe PH across etiologies in the HVPG prediction cohort.

**CSPH**	**ALD (n= 33)**	**Viral hepatitis (n= 30)**	**ALD + viral hepatitis (n=10)**	**MASLD (n=23)**	**Cholesta-tic (n=10)**	**Other (n= 22)**
**3P CSPH Probability**	0.66	0.84	0.88	0.44	0.79	0.48
**3P Predicted HVPG**	0.65	0.81	0.94	0.53	0.71	0.51
**5P CSPH Probability**	0.69	0.80	0.56	0.63	0.38	0.75
**5P Predicted HVPG**	0.71	0.70	0.50	0.68	0.62	0.68
**ANTICIPATE±NASH CSPH Probability**	0.77	0.86	1.00	0.74	1	0.79
**LSM**	0.75	0.81	1.00	0.83	0.9	0.71
**Severe PH**	**ALD (n= 33)**	**Viral hepatitis (n= 30)**	**ALD + viral hepatitis (n=10)**	**MASLD (n=23)**	**Cholesta-tic (n=10)**	**Other (n= 22)**
**3P CSPH Probability**	0.70	0.85	1.00	0.67	0.57	0.62
**3P Predicted HVPG**	0.68	0.83	1.00	0.76	0.57	0.63
**5P CSPH Probability**	0.72	0.87	1.00	0.62	0.76	0.75
**5P Predicted HVPG**	0.77	0.83	1.00	0.76	0.71	0.69
**ANTICIPATE±NASH CSPH Probability**	0.70	0.96	1.00	0.38	0.76	0.79
**LSM**	0.65	0.97	1.00	0.43	0.55	0.66

Abbreviations: AUC, area under the curve; AUROC, area under the receiver operating characteristic curve; CSPH, clinically significant portal hypertension; HVPG, hepatic venous pressure gradient; LSM, liver stiffness measurement; PH, portal hypertension, ROC, receiver operating characteristic.

Table S8. Overview of AUROCs for prediction of CSPH and severe PH in the HVPG prediction CTP A Sub-Cohort and the HVPG prediction Cohort.

	HVPG prediction CTP A Sub-cohort		HVPG prediction Cohort (CTP A/B)	
	**CSPH**	**Severe PH**	**CSPH**	**Severe PH**
**3P CSPH Probability**	0.656 (0.549 - 0.762)	0.762 (0.599 - 0.925)	0.672 (0.579 - 0.765)	0.750 (0.642 - 0.858)
**5P CSPH Probability**	0.702 (0.599 - 0.806)	0.779 (0.661 - 0.897)	0.704 (0.613 - 0.796)	0.800 (0.711 - 0.889)
**3P Predicted HVPG**	0.665 (0.559 - 0.77)	0.749 (0.573 - 0.925)	0.680 (0.587 - 0.772)	0.747 (0.635 - 0.86)
**5P Predicted HVPG**	0.690 (0.585 - 0.794)	0.782 (0.653 - 0.91)	0.699 (0.608 - 0.789)	0.793 (0.698 - 0.887)
**ANTICIPATE±NASH CSPH probability**	0.811 (0.726 - 0.895)	-	0.837 (0.766 - 0.907)	-
**LSM**	0.774 (0.682 - 0.865)	-	0.807 (0.732 - 0.882)	-

Abbreviations: AUC, area under the curve; AUROC, area under the receiver operating characteristic curve; CSPH, clinically significant portal hypertension; HVPG, hepatic venous pressure gradient; LSM, liver stiffness measurement; PH, portal hypertension, ROC, receiver operating characteristic.

Supplementary figures

Fig. S1. Study patient selection.

Abbreviations: ACLD, advanced chronic liver disease; cACLD, compensated ACLD; HCC, hepatocellular carcinoma; HVPG, hepatic venous pressure gradient; NSBB, non-selective betablocker.

Fig. S2. AUROCs for prediction of CSPH and severe Portal Hypertension.

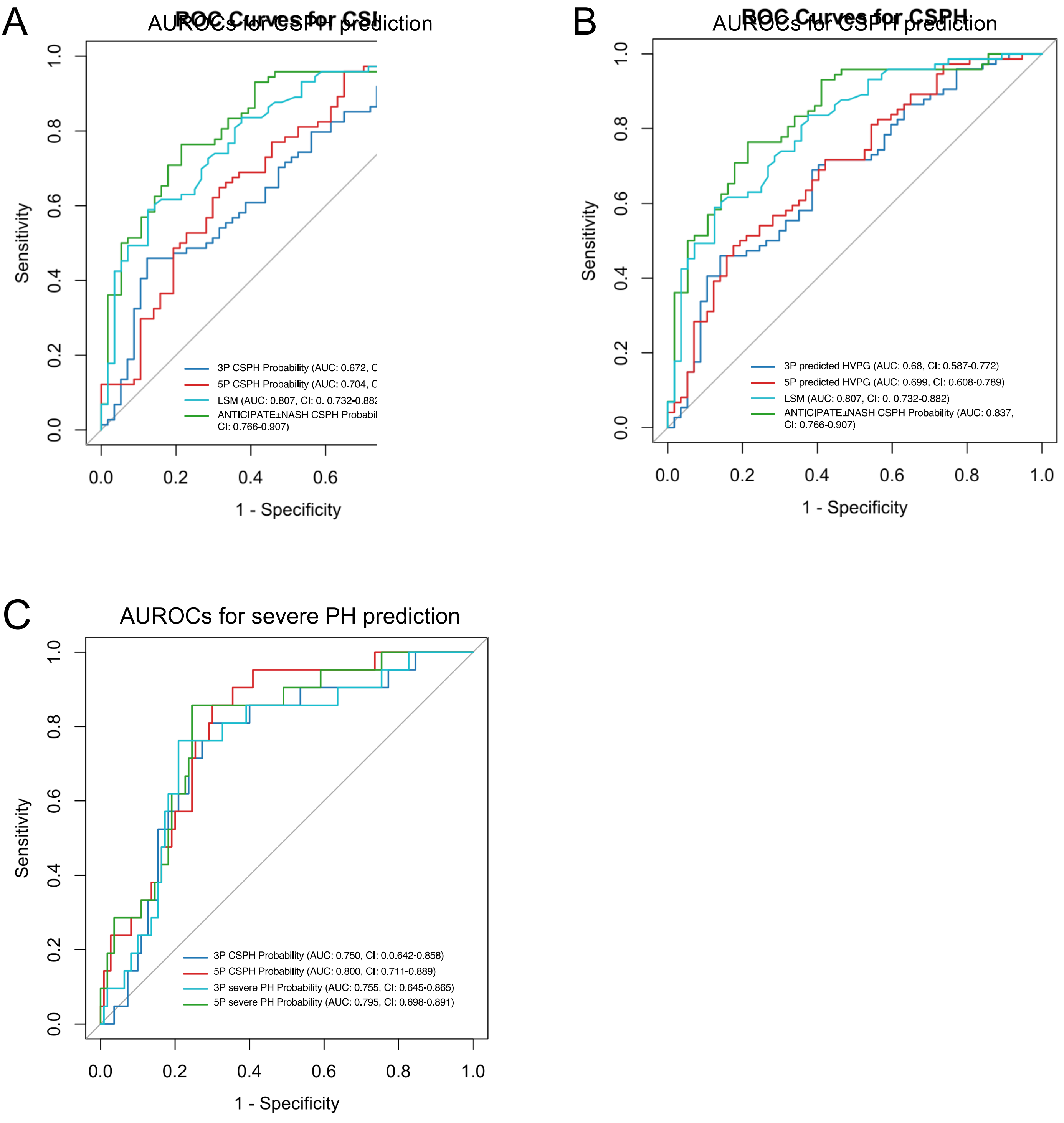

AUROCs for predicting CSPH (A, B) and severe PH (C) using Vienna 3P/5P CSPH Probability (A, C), Vienna 3P/5P predicted HVPG (B, C) as well as LSM and ANTICIPATE±NASH CSPH Probability (A, B).

Abbreviations: AUC, area under the curve; AUROC, area under the receiver operating characteristic curve; CSPH, clinically significant portal hypertension; HVPG, hepatic venous pressure gradient; LSM, liver stiffness measurement; PH, portal hypertension, ROC, receiver operating characteristic.

Fig. S3. Scatterplots showing the correlation of different NITs with HVPG in the HVPG prediction Cohort.

Scatterplots showing the correlation of 5P CSPH Probability (A), 3P CSPH Probability (B), LSM (C), 5P Predicted HVPG (D), 3P Predicted HVPG (E) and ANTICIPATE±NASH CSPH Probability (F) with HVPG in the HVPG prediction cohort. Correlation was assessed by calculation of Spearman's rank correlation coefficient.

Abbreviations: CSPH, clinically significant portal hypertension; HVPG, hepatic venous pressure gradient; LSM, liver stiffness measurement; NIT, non-invasive test.

Fig. S4. Differences in NITs between patients with and without CSPH in the HVPG prediction Cohort.

Comparison of 5P CSPH Probability (A), 3P CSPH Probability (B), LSM (C), 5P Predicted HVPG (D), 3P Predicted HVPG (E) and ANTICIPATE±NASH CSPH Probability (F) between patients with CSPH and without in the HVPG prediction cohort. Data expressed as median (IQR). NIT algorithms were compared by the Mann Whitney U test.

Abbreviations: CSPH, clinically significant portal hypertension; HVPG, hepatic venous pressure gradient; LSM, liver stiffness measurement; NIT, non-invasive test.

Fig. S5. Calibration plots displaying observed vs. predicted probabilities of CSPH by specified NITs in the HVPG prediction Cohort.

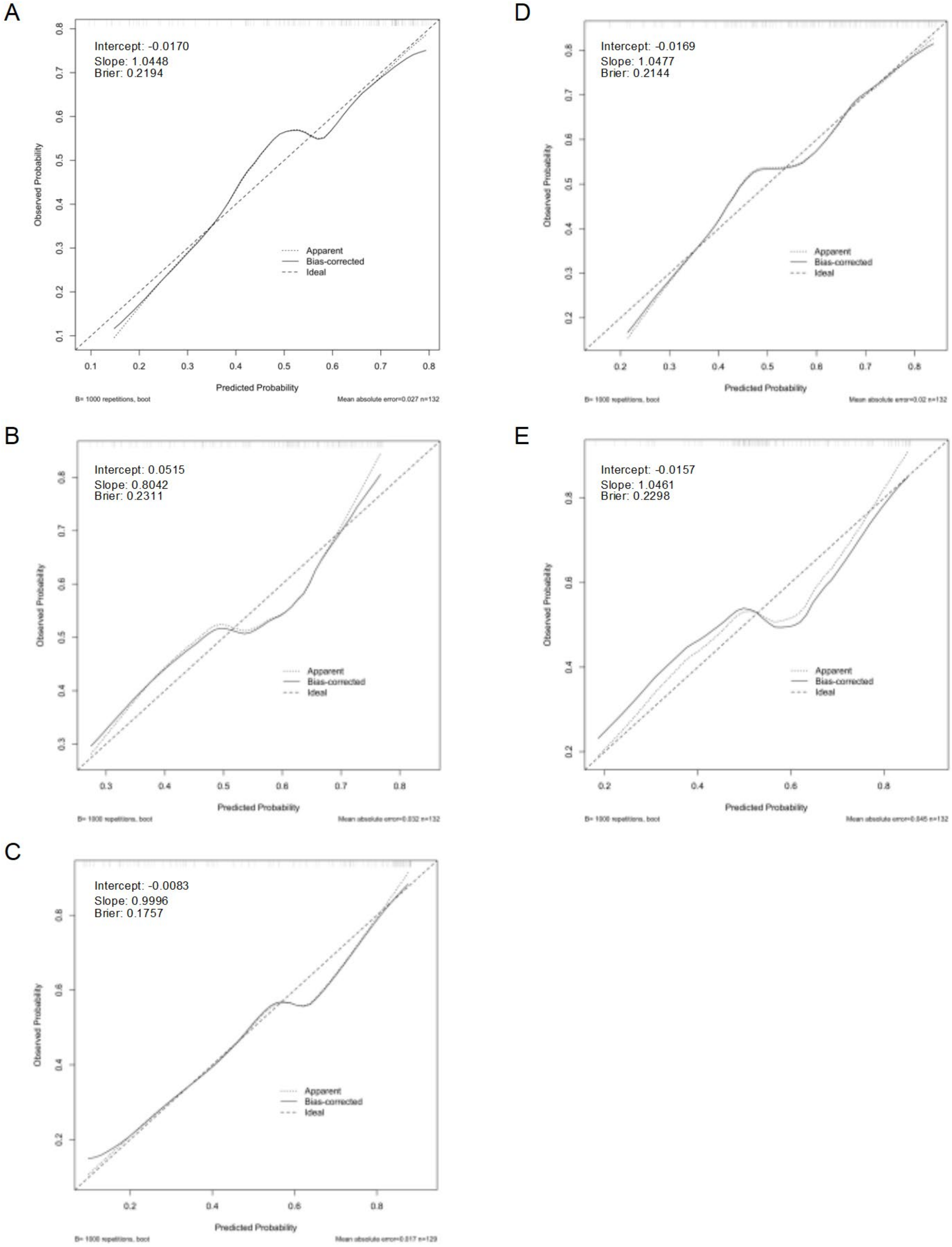

Calibration plots with bootstrapping (1,000 repetitions) displaying observed vs. predicted probabilities of CSPH by 5P CSPH Probability (A), 3P CSPH Probability (B), ANTICIPATE±NASH CSPH Probability (C), 5P predicted HVPG (D) and 3P predicted HVPG (E) in the HVPG prediction cohort.

Abbreviations: CSPH, clinically significant portal hypertension; HVPG, hepatic venous pressure gradient; NIT, non-invasive test.

Fig. S6. Time-dependent AUROCs for prediction of first hepatic decompensation in the Outcome Prediction CTP A Sub-Cohort.

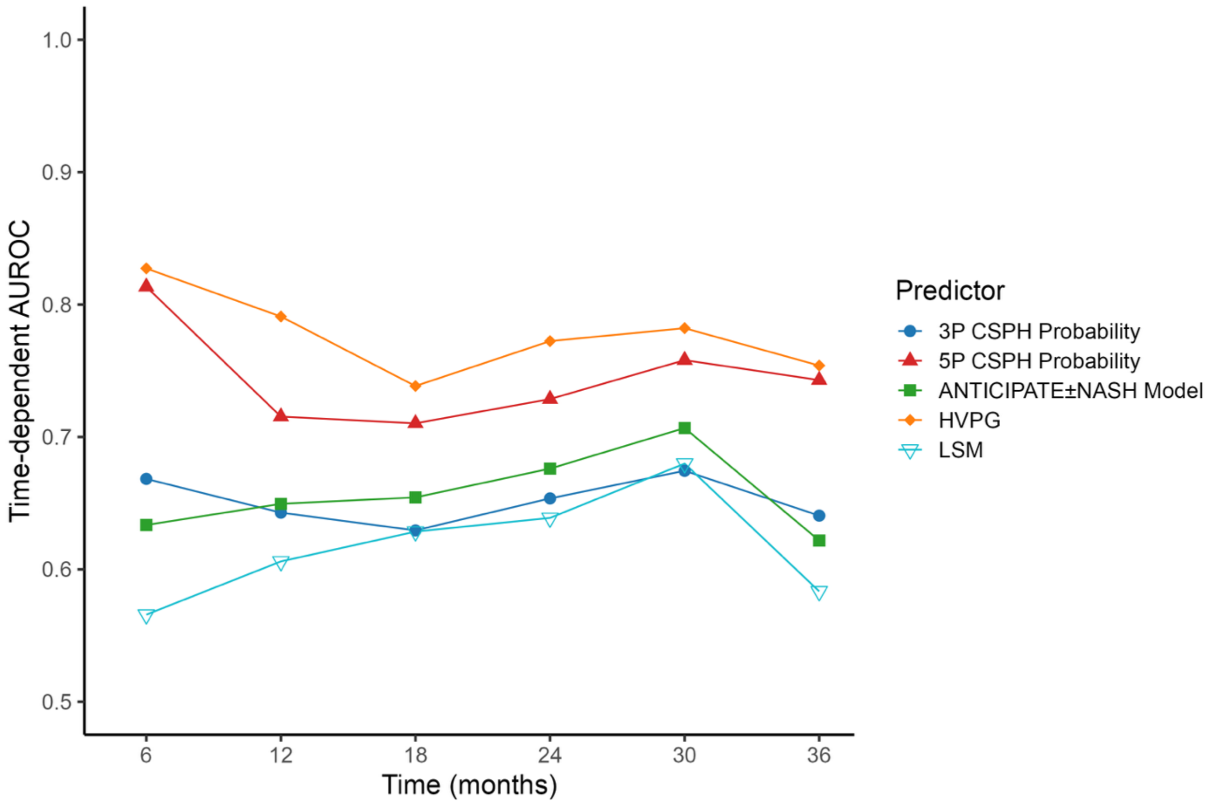

Time-dependent AUROCs for prediction of first hepatic decompensation by the Vienna 3P and 5P CSPH probabilities as well as HVPG, LSM and ANTICIPATE±NASH CSPH probability.

Abbreviations: AUC, area under the curve; AUROC, area under the receiver operating characteristic curve; CSPH, clinically significant portal hypertension; HVPG, hepatic venous pressure gradient; LSM, liver stiffness measurement; ROC, receiver operating characteristic.

Fig. S7. Cumulative incidence plots for first hepatic decompensation as stratified by the Vienna 3P/5P predicted HVPG to identify patients at high risk of decompensation in the Combined Vienna and Hannover Cohorts.

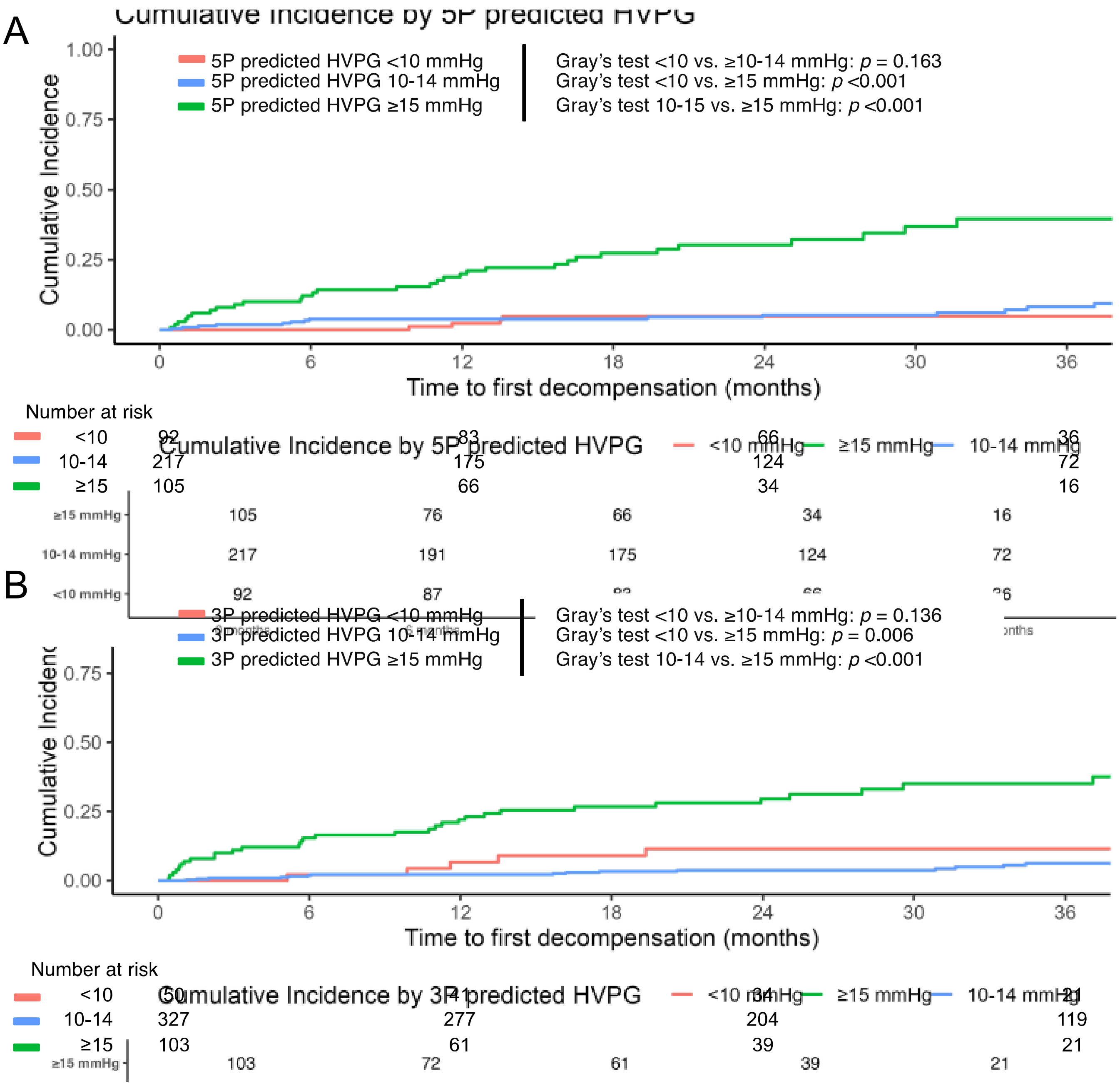

Cumulative incidence of first hepatic decompensation as stratified by 5P predicted HVPG (A) and 3P predicted HVPG (B). Occurrence of hepatocellular carcinoma or non-liver related death were treated as competing events. The cumulative incidences were compared using Gray's test. Abbreviations: CSPH, clinically significant portal hypertension; HVPG, hepatic venous pressure gradient; LSM, liver stiffness measurement.

Fig. S8. Cumulative incidence plots for first hepatic decompensation as stratified by proposed decision rules to identify patients at high risk of decompensation in the Outcome Prediction CTP A Sub-Cohort.

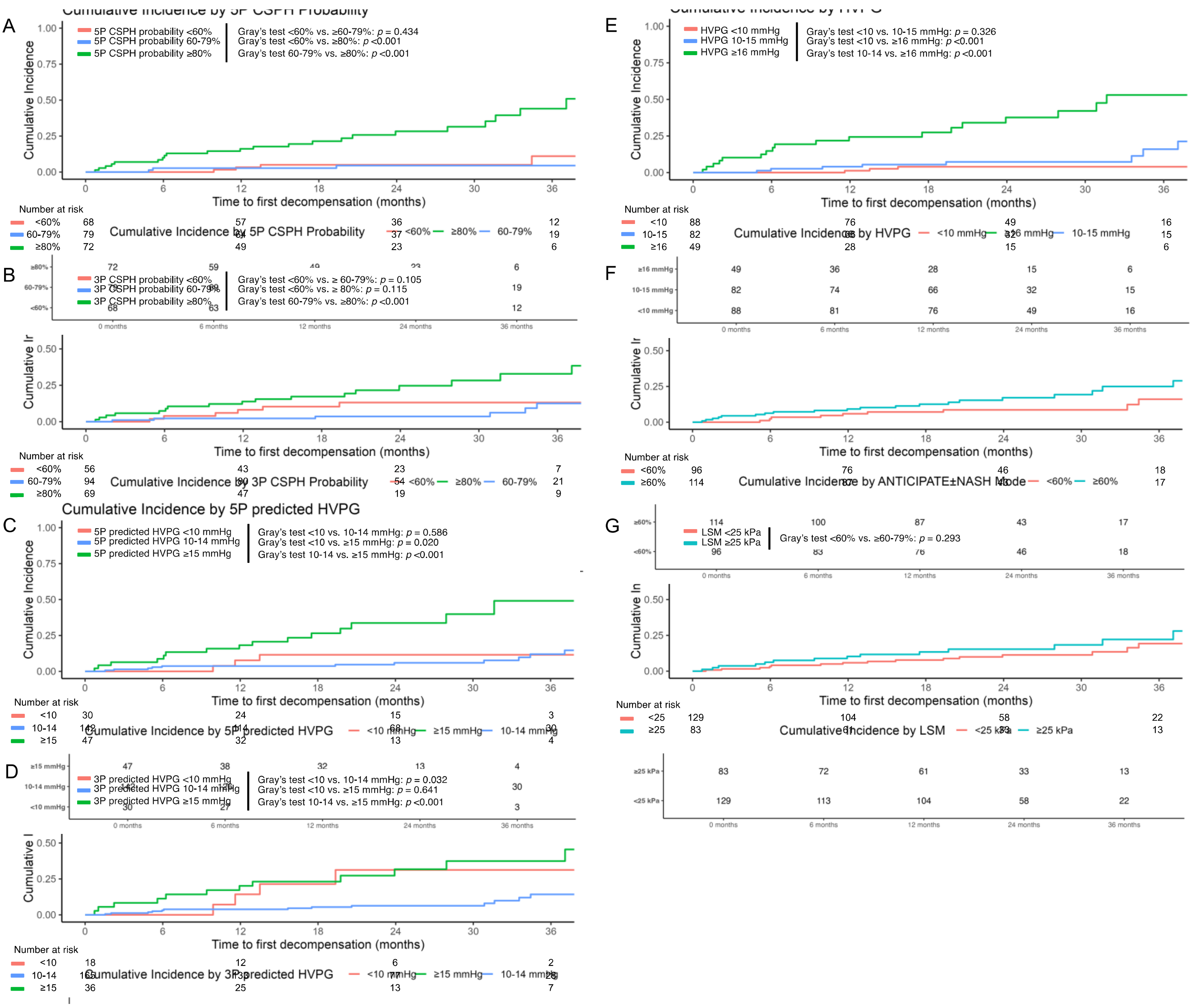

Cumulative incidence of first hepatic decompensation as stratified by 5P CSPH Probability (A), 3P CSPH Probability (B), 5P predicted HVPG (C), 3P predicted HVPG (D), HVPG (E), ANTIICPATE±NASH (F) and LSM (G). Occurrence of hepatocellular carcinoma or non-liver related death were treated as competing events. The cumulative incidences were compared using Gray's test. Abbreviations: CSPH, clinically significant portal hypertension; HVPG, hepatic venous pressure gradient; LSM, liver stiffness measurement.

Fig. S9. Cumulative incidence of first hepatic decompensation in patients with and without etiological cure in the Vienna Outcome Prediction Cohort.

Cumulative incidence of first hepatic decompensation in the subgroups of patients with etiological cure (Cure) and those without (No Cure) in the Vienna Outcome prediction cohort. Patient subgroups are displayed as stratified by 5P CSPH probability (A, E), 3P CSPH probability (B, F), 5P predicted HVPG (C, G) and 3P predicted HVPG (D, H). Occurrence of hepatocellular carcinoma or non-liver related death were treated as competing events. The cumulative incidences were compared using Gray’s test. Abbreviations: CSPH, clinically significant portal hypertension; HVPG, hepatic venous pressure gradient.

Fig. S10. AUROCs for prediction of severe Portal Hypertension by 3P/5P CSPH and severe PH probabilities in the HVPG prediction Cohort.

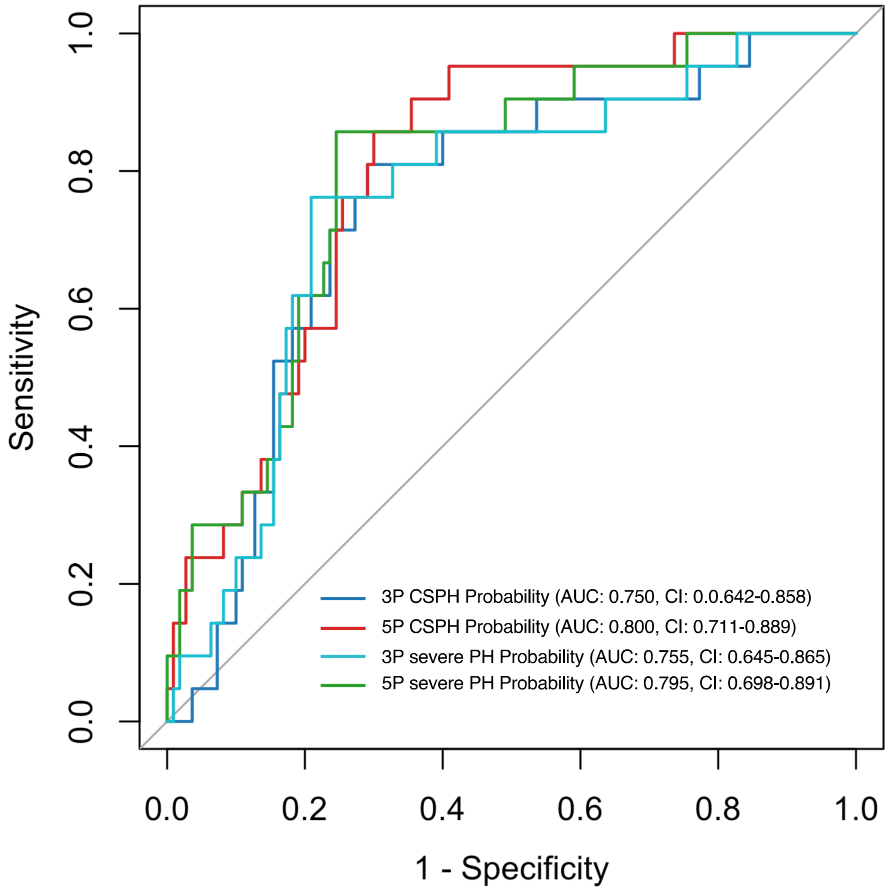

AUROCs for predicting severe PH using Vienna 3P/5P CSPH and severe PH probabilities.

Abbreviations: AUC, area under the curve; AUROC, area under the receiver operating characteristic curve; CSPH, clinically significant portal hypertension; HVPG, hepatic venous pressure gradient; LSM, liver stiffness measurement; PH, portal hypertension, ROC, receiver operating characteristic.

Fig. S11. Graphical depiction of the association between specified predictors with the Log Odds of CSPH.

Graphical depiction of the association between 5P CSPH Probability (A), 3P CSPH Probability (B), ANTICIPATE±NASH CSPH Probability (C), 5P predicted HVPG (D) and 3P predicted HVPG (E) and the log odds of CSPH. The yellow line indicates a linear fit, treating each predictor as continuous and linear, whereas the blue line represents the predictor modeled non-linearly using restricted cubic splines with four degrees of freedom. Incorporating a non-linear functional form did only significantly enhance the overall goodness-of-fit for the 3P predicted HVPG (likelihood ratio test, $p = 0.015$). Hence, for calibration analysis a non-linear (spline) CPSH model derived from 3P predicted HVPG was used.

Abbreviations: CSPH, clinically significant portal hypertension.
